# Functional RNAi Screening Identifies G2/M and Kinetochore Components as Modulators of TNFα/NF-κB Prosurvival Signaling in Head and Neck Squamous Cell Carcinoma

**DOI:** 10.1158/2767-9764.CRC-24-0274

**Published:** 2024-11-07

**Authors:** Ethan L. Morgan, Anthony D. Saleh, Shaleeka Cornelius, Sophie G. Carlson, Tiffany Toni, Hui Cheng, Jun Jeon, Ramya Viswanathan, Xinping Yang, Christopher Silvin, Paul E. Clavijo, Anastasia L. Sowers, James B. Mitchell, Pinar Ormanoglu, Madhu Lal Nag, Scott E. Martin, Zhong Chen, Carter Van Waes

**Affiliations:** 1 Tumor Biology Section, Head and Neck Surgery Branch, National Institute on Deafness and Communication Disorders, National Institutes of Health, Bethesda, Maryland.; 2 School of Life Sciences, University of Sussex, Brighton, United Kingdom.; 3 Radiation Biology Branch, Center for Cancer Research, National Cancer Institute, Bethesda, Maryland.; 4 RNAi Screening Facility, National Center for Advancing Translational Sciences, National Institutes of Health, Bethesda, Maryland.

## Abstract

Immune and radiation resistance of cancer cells to cytotoxicity mediated by TNFα is promoted by the transcription factor NF-κB in several cancers, including head and neck squamous cell carcinoma (HNSCC). Genomic alterations that converge on the TNFα/NF-κB signal axis were found in ∼40% of HNSCCs by The Cancer Genome Atlas. However, identification of therapeutic targets that contribute to aberrant TNFα/NF-κB activation and resistance has been challenging. Here, we conducted a functional RNAi screen to identify regulators of TNFα-induced NF-κB activation and cell viability, using parallel NF-κB β−lactamase reporter and cell viability assays in a HNSCC cell line which harbors expression and genomic alterations typically found in human papillomavirus–negative HNSCC. Besides multiple components of canonical TNFα/NF-κB signaling, we identified components of the WNT, NOTCH, and TGFβ pathways that we previously showed contribute to noncanonical activation of NF-κB. Unexpectedly, we also observed that multiple G2/M cell-cycle kinases [Aurora kinase A, polo-like kinase 1, WEE1, and threonine tyrosine kinase (TTK)], and structural kinetochore/microtubule components (NDC80 and NUF2), modulate TNFα-induced NF-κB activation and cell viability. Several of these targets inhibit TNF-induced nuclear translocation of RELA, consistent with prior reports linking NF-κB activation to G2/M kinases or microtubule assembly. Further investigation of an understudied mitotic kinase, TTK/monopolar spindle 1, shows that its inhibition or depletion attenuates TNFα-induced RELA nuclear translocation, promoting cell death, DNA damage, polyploidy, and mitotic catastrophe, leading to radiosensitization. Together, our RNAi screening identifies a critical linkage between the G2/M cell-cycle checkpoint/kinetochore components and NF-κB activity, as well as targets that can sensitize HNSCC cells to TNFα or radiation.

**Significance:**

Here, RNAi library screening reveals that multiple G2/M and kinetochore components, including TTK/monopolar spindle 1, modulate TNFα-induced NF-κB activation, cell survival, and genotoxicity, underscoring their potential importance as therapeutic targets in HNSCC.

## Introduction

TNFα is an important mediator of immune cytotoxicity in cancer (1). Radiotherapy can also induce TNFα and cell death via TNF receptor (TNFR) or via cell death pathways activated by reactive oxygen species (ROS) and genotoxic DNA damage (2). However, the cytotoxic effects of TNFα and genotoxic therapies can be attenuated via signal activation of the canonical IKK α/β/γ complex, which promotes phosphorylation and degradation of inhibitor kappa B, nuclear translocation, and activation of the prosurvival transcription factor NF-κB1/RELA (3). IKK and NF-κB1/RELA induce transcription and increased expression of antiapoptotic proteins, which can enhance resistance to caspase and mitochondrial cell death (3). We have shown that the canonical IKK–NF-κB/RELA pathway is often aberrantly activated and promotes resistance to effects of TNFα, as well as immune-mediated and radiation cytotoxicity, in head and neck squamous cell carcinomas (HNSCC; refs. 4–6).

HNSCCs affect ∼900,000 new patients and cause ∼450,000 deaths annually and are the sixth most common cancer worldwide (7). HNSCCs include a subset linked to infection with high-risk human papillomaviruses (HPV^+^), and those linked to tobacco and alcohol use (HPV^−^; ref. 7). HPV^+^ HNSCCs have a better prognosis and survival when compared with HPV^−^ HNSCCs, in patients receiving standard treatment. Significantly, The Cancer Genome Atlas (TCGA) uncovered genomic alterations that encode proteins that form critical components of the TNFα receptor–NF-κB/death domain receptor signaling complex, which is deregulated and implicated in cell survival and therapeutic resistance in cancer (8). These include amplifications of chromosome 11q13/22 that harbor genes Fas-associated death domain and baculovirus inhibitor of apoptosis repeat containing (BIRC2/cIAP1) in ∼30% of HPV^−^ HNSCCs. A mutually exclusive subset harboring mutations of caspase 8 affects an additional 10% of HPV^−^ cases. Furthermore, deletions in TNF-associated factor 3 (TRAF3) and overexpression of BIRC3 (cIAP2) are detected in ∼20% of HPV^+^ HNSCCs (8). IAP1/2 are ligases that form a polyubiquitin scaffold linking the TNFR complex to activate the canonical IKK/NF-κB prosurvival pathway, while antagonizing caspase-mediated cell death (9). TRAF3 loss results in stabilization of the NF-κB–inducing kinase (10). Furthermore, our pan-cancer comparison of TCGA data reveals that HNSCC and other squamous cancers harbor the highest frequency of genomic alterations in these TNFα/NF-κB/cell death pathways (11). Additionally, HPV itself induces NF-κB activity via the E6 oncogene (12). Thus, the activation of the NF-κB pathway via genomic alteration or viral induction makes it a potentially important target for therapy (13). However, as IKK and NF-κB–inducing kinase knockout or inhibitors have demonstrated significant toxicity (14–16), identification of other suitable therapeutic targets that inhibit aberrant activation of TNFα–NF-κB prosurvival signaling and enhance cancer cell cytotoxicity without such undesirable toxicities has been challenging.

To screen for modulators of TNFα-induced NF-κB prosurvival signaling, we developed an HPV^−^ HNSCC cell line stably expressing NF-κB promoter response elements linked to a β-lactamase reporter gene (5) and performed an RNA interference (RNAi) screen for targets resulting in inhibition of TNFα-induced NF-κB activation, and cell viability. This revealed that siRNAs targeting multiple components of the G2/M cell-cycle checkpoint, kinetochore, and microtubules inhibit TNFα-induced NF-κB nuclear translocation, activation, and cell survival. These include several potential druggable targets, such as the kinases polo-like kinase 1 (PLK1), Aurora kinase A (AURKA), WEE1, and threonine tyrosine kinase/monopolar spindle 1 (TTK; referred to as TTK from hereon). In this report, we further demonstrate that TTK modulates NF-κB activity in HNSCC and contributes to TNFα and radiation resistance.

## Materials and Methods

### HNSCC cell lines

The UMSCC1 (HPV^−^) and UMSCC47 (HPV^+^) cell lines, from the University of Michigan squamous cell carcinoma (UMSCC) series, were obtained from Dr. T.E. Carey. These UMSCC cell lines were authenticated by genotyping with nine markers and whole-exome sequencing as described previously (17, 18). UMSCC cell lines were cultured in minimal essential medium (MEM) supplemented with 10% FCS, penicillin and streptomycin (100 μg/mL), and MEM nonessential amino acids. The cell line stocks were preserved frozen in liquid nitrogen and used within 3 months of culture.

### *In vitro* RNAi screening

The generation and characterization of the UMSCC1^κB^ reporter line were previously described (5). For screening, siRNA libraries (Ambion Silencer Select libraries V4, Thermo Fisher Scientific) plated in 384 well plates were reverse transfected using Lipofectamine in MEM into 1,500 UMSCC1^κB^ cells/well, and cultured for 56 hours followed by 16 hour with TNFα (20 ng/mL; see schema, Supplementary Fig. S1A). NF-κΒ activation (blue) and viability (green) were then simultaneously quantified via β-lactamase FRET assay, to identify siRNAs inhibiting NF-κB activity and/or cell viability, and the effects on viability were independently verified by a parallel CellTiter-Glo (CTG) assay. We performed two replicate screens of a kinome siRNA library which each contains siRNAs targeting 709 kinase mRNAs and a druggable genome (DG) screen targeting 9,031 mRNAs (Supplementary Table S1); and a whole genome (WG) screen targeting 21,584 mRNAs (Supplementary Table S2). Each library includes three unique siRNAs targeting different regions of the same mRNA. The replicate kinome library screens 1 versus 2 were highly correlated as measures of library and assay reproducibility (Supplementary Fig. S2A). The viability as measured by green FRET activity (Supplementary Fig. S2B) and green FRET versus CTG (K1 vs. CTG, *r* = 0.31 K2 vs. CTG, *r* = 0.419) assays was also correlated in linear regression analyses. For the RNAi screens, median absolute deviation (MAD), redundant siRNA analysis (RSA), or common seed analysis (CSA) methods were used to integrate and limit false positives for the results of multiple siRNAs tested for the same gene (19). For validation, 333 candidates significantly modulating NF-κB in primary WG screen by CSA, RSA, and potentially related to NF-κB or cell cycle by Ingenuity Pathway Analysis (IPA) were selected for secondary screen with three independent siRNAs from the Ambion Silencer V3 druggable and whole extension libraries, and CSA of six siRNAs from the primary and secondary screens was used to determine the significance and prioritize hits most likely due to on-target effects (Supplementary Table S3; ref. 20).

### Statistical analysis

IPA (SCR_008653) was performed to classify the top pathways significantly modulated along with NF-κB reporter activity by depletion of the 769 candidates from the WG screen ranked by CSA (Supplementary Table S4). Gene expression data and patient progression-free survival (PFS) were downloaded from the Supplementary Information of the pan-squamous cancer TCGA study (18). Patient overall survival (OS) data were downloaded from Firehose at the Broad Institute (https://confluence.broadinstitute.org/display/GDAC/Home/). For differential gene expression analysis between normal and tumor tissue, and HPV^−^ and HPV^+^ tumor tissue, Wilcoxon sum rank test was used to assess statistical significance. The clinical survival endpoints for patients were PFS and OS. The PFS and OS curves were obtained using the Kaplan–Meier method and were compared using the log-rank test. The Cox proportional hazards model was used to estimate HRs with 95% confidence intervals. To examine if the relationship between TTK expression, tumor stage, or treatment and survival in 434 HPV^−^ and 80 HPV^+^ tumors was significant, we performed a multivariate analysis. TTK expression was dichotomized as high or low using an outcome-orientated method that provides a value for a cut point that corresponds to the most significant relationship with survival (surv cutpoint function). The tumor stage was grouped to Stage_Low (stages I and II) and Stage_High (stage III and stages IVA, IVB, and IVC). For treatment, as all patients for which tissue was submitted to the TCGA had surgery, we controlled for surgery only (no radiation) and surgery and radiation (with radiation).

The combination index (CI) for TTK inhibitor with combinations on cell viability was analyzed for synergy (CI < 1) or additive (CI ∼ 1) effects using the Chou–Talalay method using CompuSyn (21). All graphs were prepared using the GraphPad Prism (RRID: SCR_002798; GraphPad) or R software version 4.0.2. Error bars represent mean ± SE. Statistical significance was determined as follows: NS, not significant; *, *P* < 0.05; **, *P* < 0.01; ***, *P* < 0.001.

### Data availability

All data are included within the main text and supplementary files.

## Results

### RNAi screening identifies canonical and noncanonical signal pathway, G2/M kinases, and kinetochore components as modulators of TNFα-induced NF-κB reporter activity and cell viability

**Figure 1:**
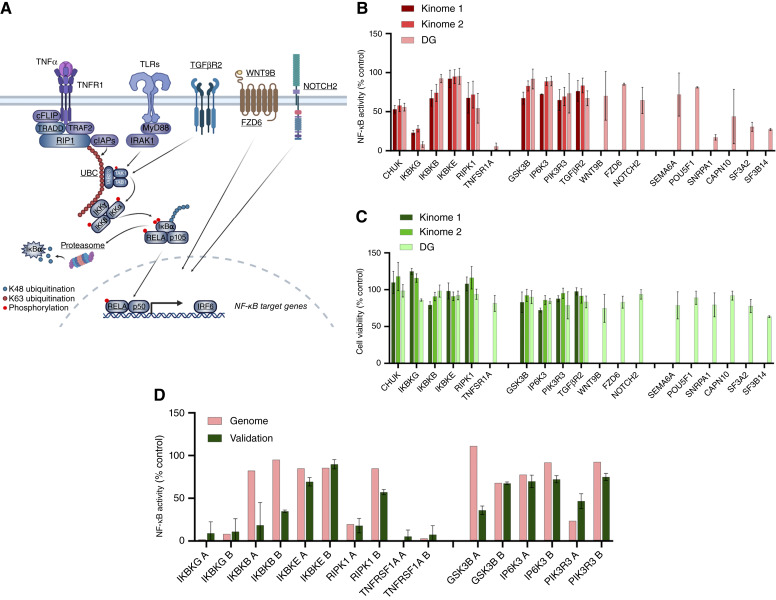
RNAi screening identifies G2/M kinases and kinetochore components as modulators of TNFα-induced NF-κB reporter activity and cell viability. **A,** Schematic demonstrating identified canonical and noncanonical regulators of NF-κB from the RNAi screening. Identified hits are in bold and underlined. **B,** NF-κB reporter activity in UMSCC1^κB^ after transfection with select siRNAs from the kinome 1, kinome 2, and DG RNAi screens. Canonical and noncanonical hits are shown. NF-κB activity is shown as a percentage of a negative control siRNA (set at 100%). **C,** Cell viability in UMSCC1^κB^ after transfection with select siRNAs from the kinome 1, kinome 2, and DG RNAi screens. Canonical and noncanonical hits are shown. Cell viability is shown as a percentage of a negative control siRNA (set at 100%). **D,** Validation of NF-κB reporter activity in UMSCC1^κB^ comparing primary and secondary screen with three independent siRNAs. Select siRNAs from the RNAi screen are shown compared with the DG results (Genome in figure). Canonical and noncanonical hits are shown. NF-κB activity is shown as a percentage of a negative control (set at 100%). Each RNAi screen contained three individual siRNA targeting each gene. Validation siRNA experiments used separate siRNAs not used in the RNAi screens. (**A,** Created with BioRender.com.)

We previously established the HPV^−^ HNSCC cell line UMSCC1^κΒ^, which stably expresses an NF-κB response element–β-lactamase reporter gene (5). This cell line was then used to screen for modulators of TNFα-induced NF-κB activity and cell viability as described (Supplementary Fig. S1A and S1B; ref. 5). To assess the reproducibility of the assay, we first performed and compared the results of two separate screens with an siRNA library (Ambion Gene Silencer Kinome v4) containing siRNAs targeting 709 kinase mRNAs and assessed the effects on NF-κB activity and cell viability. Our data confirmed a high degree of correlation between the effects of knockdown of these targets on NF-κB activity or cell viability, validating the robust global reproducibility between the screens (kinome 1 vs. kinome 2; Supplementary Table S1A; Supplementary Fig. S2A and S2B). For prioritization, we used a previously standardized approach employing the MAD (a cutoff of −1.5 MAD), or alternatively, the redundant siRNA analysis (RSA) and log *P* values (a cutoff of −1.96 log P) for siRNAs targeting the same mRNA, to rank those reducing NF-κB activity and/or cell viability (Supplementary Table S1A; refs. 19, 22). Supporting the functional validity of the screen, the top 27 kinases whose knockdown inhibited NF-κB activity and/or cell viability (MAD < −1.5 or logP < −1.96) included components of the TNFR–IKK–NF-κB pathway [e.g., *RIPK1*, *IKBKG* (IKKγ), *CHUK* (IKKα), and *IKBKB* (IKKβ; Supplementary Table S1A; Fig. 1A–C)]. A third independent screen using a broader DG siRNA library targeting >10K mRNAs overlapping the kinome library also showed significant inhibition of NF-κB and/or cell viability by knockdown of many of the same plus additional components of the TNFR–IKK–NF-κB and proteasome pathway (e.g., *TNFRSF1A*, multiple *PSMA/B* genes, *RELA*; Fig. 1A–C; Supplementary Table S1B). Other targets included components of TGFβ, NOTCH, PI3K, WNT/FZD/GSK3, and SEMA6A signaling, several of which have been implicated in modulating NF-κB activity in HNSCC or other contexts (Supplementary Tables S1A and S1B; Fig. 1A–C; refs. 23–27).

Having demonstrated the reproducibility and functional utility of the assay, we next screened a WG siRNA library targeting 21,567 mRNAs overlapping the kinome and DG libraries for inhibitors of NF-κΒ activation, using the same conditions described in the previous screens (Supplementary Table S2A). To further minimize false positives caused by off-target effects of siRNAs with similar seed regions in the first seven to eight bases of the siRNA, CSA correction was also performed (Supplementary Table S2B), as previously described (20). The screening data for each CSA-corrected mRNA were ranked by log (*P* value) for inhibition of NF-κΒ activity, and a broad data threshold was set at −2 log (*P* value), which yielded 769 mRNAs for which siRNAs significantly inhibited NF-κΒ activity (Supplementary Table S2B). This WG ranked list also included many of the known canonical signaling molecules in the TNFR complex, downstream IKKs, proteasome, and NF-κΒ transcription factor subunits and reported noncanonical modulators of NF-κB. A subset of these selected for further validation by three independent siRNAs demonstrated similar or greater inhibition when compared with those in the WG library (Fig. 1D; Supplementary Table S3).

**Figure 2:**
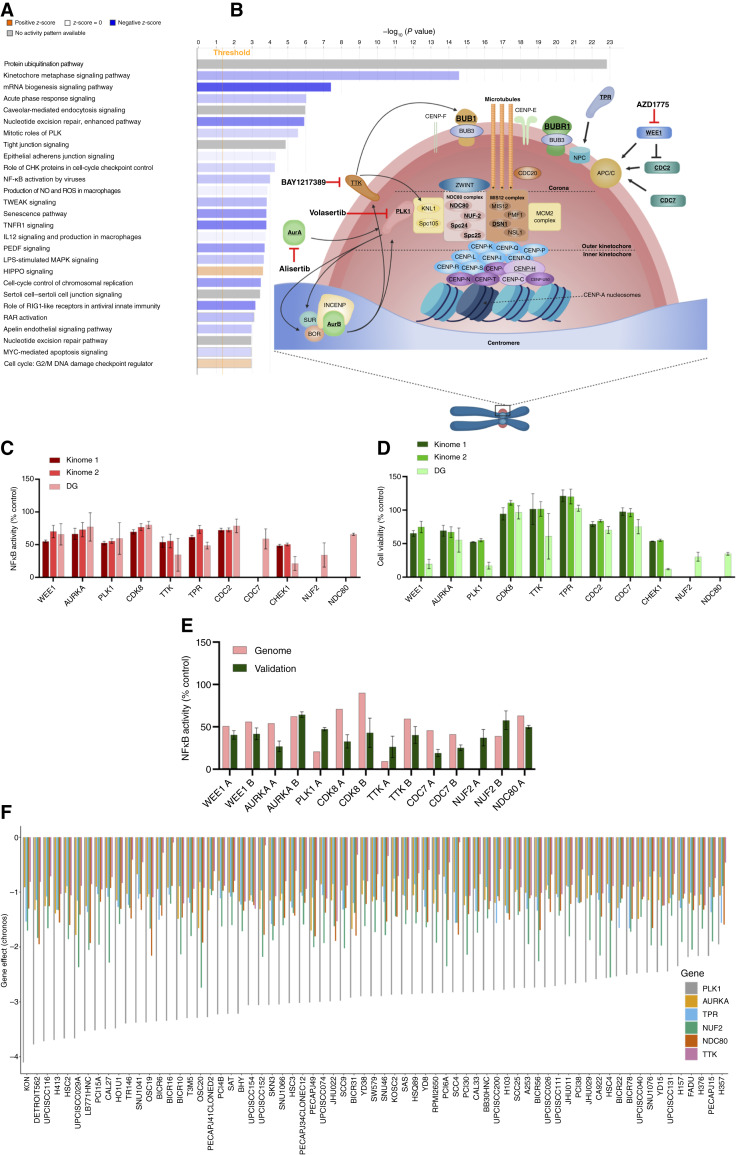
Identification of G2/M checkpoint and kinetochore components as modulators of TNF-induced NF-κB activity in HNSCC cells. **A,** IPA was performed using data for the 769 candidates for which depletion significantly modulating NF-κB activity and ranked as < −2 LogP reporter seed corrected by CSA (Supplementary Table S2). **B,** Schematic demonstrating identified G2/M checkpoint kinases and kinetochore components significantly modulating NF-κB activity in UMSCC1^κB^ cells. Identified hits are in bold and underlined. **C,** NF-κB reporter activity in UMSCC1^κB^ after transfection with select siRNAs from the kinome 1, kinome 2, and DG RNAi screens. NF-κB activity is shown as a percentage of a negative control siRNA (set at 100%). **D,** Cell viability in UMSCC1^κB^ after transfection with select siRNAs from the kinome 1, kinome 2, and DG RNAi screens. Cell viability is shown as a percentage of a negative control siRNA (set at 100%). **E,** Validation of NF-κB reporter activity in UMSCC1^κB^ after transfection with independent siRNAs. Select siRNAs from the secondary RNAi screen are shown compared with the DG results (genome in figure). NF-κB activity is shown as a percentage of a negative control (set at 100%). Each RNAi screen contained three individual siRNA targeting each gene. Validation siRNA experiments used separate siRNAs not used in the RNAi screens. **F,** Chronos-inferred gene dependency scores for PLK1, AURKA, NUF2, NDC80, and TTK in 69 head and neck cancer cell lines in the CRISPR knockout screens from Broad’s Achilles and Sanger’s Score projects. The order is based on the gene effect of PLK1 [most dependent cell line (lowest value) to least dependent cell line (highest value); **B,** created with BioRender.com.]

Pathway classification of the 769 WG screen components for which knockdown significantly decreased NF-κΒ activity was performed using IPA, which independently identified several significantly modulated pathways previously implicated in modulating NF-κB, such as protein ubiquitination (e.g., proteasome), acute phase response, TWEAK, and TNFR1 signaling (Fig. 2A; Supplementary Table S4). Unexpectedly, several classifiers related to mitosis, the G2/M checkpoint, and related kinases were also identified, including kinetochore metaphase signaling, mitotic roles of PLKs, and G2/M DNA damage checkpoint response (Fig. 2A and B). The review of our independent kinome and DG screening assays above also confirmed that the knockdown of multiple G2/M kinases, cell cycle, kinetochore, and tubulin components reproducibly inhibited NF-κB activity and cell viability (e.g., WEE1, PLK1, AURKA, TTK, TPR, NUF2, NDC80, BUB1B, and TUBA1B; Fig. 2C and D; Supplementary Tables S1 and S2). Many of these showed consistent inhibition of NF-κB activity across repeated screens in which the kinome, DG, and WG libraries each included the same three siRNAs targeting their corresponding mRNA (Fig. 2C and D). From the genes significantly ranked by CSA and/or RSA and including known canonical and noncanonical NF-κB, G2/M, and kinetochore components annotated by IPA, we selected 333 for further validation. The results for three primary WG plus three secondary independent siRNAs were used for CSA and ranking by *z*-score (Supplementary Tables S2 and S3). We observed similar or greater NF-κB inhibition for many of those detected in the WG and validation screen (e.g., WEE1, PLK1, AURKA, TTK, TPR, NUF2, NDC80, BUB1B, and TUBA1B; Figs. 1D and 2E).

To further examine the relative dependence of HNSCC upon several of these G2M/kinetochore components for cell survival, we used the DepMap portal, which contains the CRISPR knockout screens from Broad’s Achilles and Sanger’s Score projects in 68 head and neck cancer cell lines. Negative scores imply cell growth inhibition and/or death following gene knockout. Gene effect scores were inferred by Chronos; scores are normalized such that nonessential genes have a median score of 0 and independently identified common essentials have a median score of −1 (28). We used this database to assess the dependence of a large panel of head and neck cancer cell lines on the expression of PLK1, AURKA, TPR, NUF2, NDC80, and TTK. The data demonstrate that PLK1 and NUF2 are the most essential genes among these cancers, with the lowest Chronos scores. Among the other genes, NDC80, 93% of the 69 cell lines have Chronos scores of < −1; TPR, 70% of the cell lines have scores of < −1; AURKA, 61% of the cell lines have scores of < −1. TTK exhibited values below 0, demonstrating a variable level of dependency in HNSCC cells (Fig. 2F). These data are consistent with our recent work, in which we established a novel interaction and role for the checkpoint kinase WEE1 in TNFα–IKK–NF-κB activation and prosurvival signaling in HNSCC (5). Our overall screening data suggest a broader repertoire of G2/M kinases and kinetochore components can modulate NF-κB activity and/or viability in HNSCC cells.

### Mitotic kinases and kinetochore components modulate NF-κB nuclear translocation, activity, cell viability, and TNFα resistance in HNSCC cells

**Figure 3:**
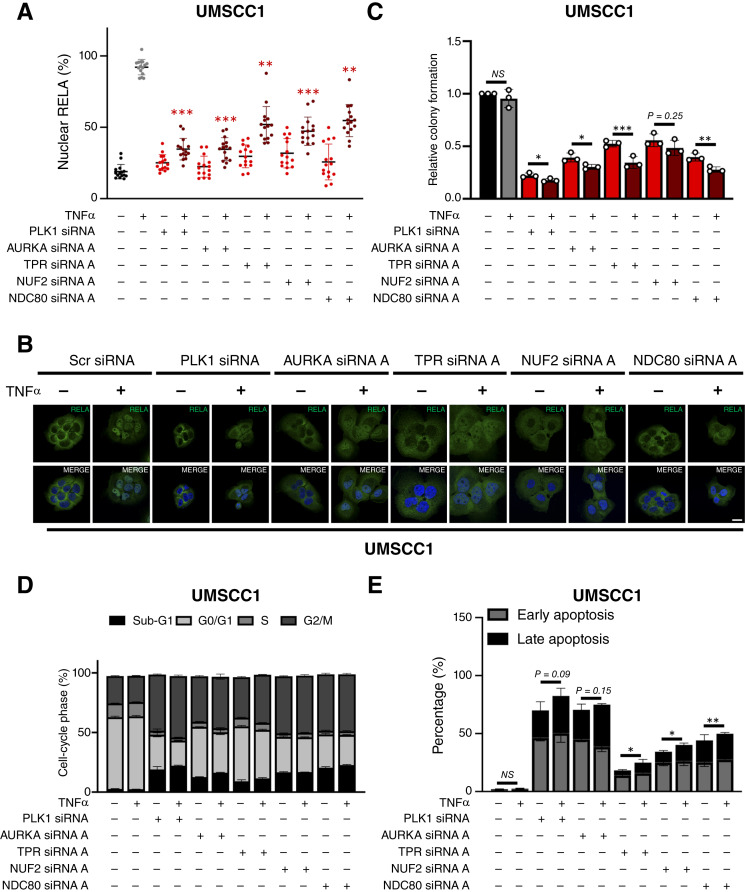
Knockdown of G2/M kinases and kinetochore proteins inhibits NF-κB activity and sensitizes HNSCC cells to TNF-induced cell death. **A,** Quantification of percentage nuclear RELA in UMSCC1 cells transfected with siRNA targeting PLK1, AURKA, TPR, NUF2, and NDC80 for 72 hours, with TNFα (20 ng/mL) added for the final 30 minutes. DAPI (4′, 6-diamidino-2-phenylindole) was used as a nuclear counterstain. Scale bar, 10 μmol/L. Data represent the percentage nuclear localization of RELA from 15 cells and were analyzed using ImageJ as previously (12). **B,** Representative immunofluorescence images of the data in **A**. **C,** Colony formation assay of UMSCC1 cells transfected with siRNA targeting PLK1, AURKA, TPR, NUF2, and NDC80 for 72 hours, with TNFα (20 ng/mL) added for the final 24 hours. DAPI was used as a nuclear counterstain. **D,** Cell-cycle analysis of UMSCC1 cells transfected with siRNA targeting PLK1, AURKA, TPR, NUF2, and NDC80 for 72 hours, with TNFα (20 ng/mL) added for the final 24 hours. Statistics are shown in Supplementary Table S5. **E,** Annexin V analysis of UMSCC1 cells were transfected with siRNA targeting PLK1, AURKA, TPR, NUF2, and NDC80 for 72 hours, with TNFα (20 ng/mL) added for the final 24 hours. Bars represent the means ± SD. All experiments are representative of at least three biological replicates. NS, not significant; *, *P* < 0.05; **, *P* < 0.01; ***, *P* < 0.001.

To validate the functional significance of G2/M components identified by our screens, we performed additional experiments to confirm the role of several identified candidates in the modulation of NF-κB activation and cell viability in HNSCC cells. Because prior reports have implicated a few individual kinases (PLK1 and AURKA) and microtubule assembly in modulating NF-κB activation and/or cytoplasmic-nuclear translocation (29–35), we analyzed the effects of depletion of several kinase and kinetochore components on TNFα-induced RELA nuclear localization, an essential step involved in canonical IKK-dependent NF-κB activation, using immunofluorescence confocal microscopy. We first confirmed depletion of the corresponding proteins for the mitotic kinases PLK1, AURKA, and TPR, as well as the structural kinetochore components NUF2 and NDC80, in the parental HPV^−^ UMSCC1 line used for the reporter line in our screen (Supplementary Fig. S3A–S3E). Remarkably, depletion of each component significantly reduced TNF-induced RELA nuclear translocation (Fig. 3A and B), consistent with our NF-κB reporter data. We next investigated the impact of depletion of these components on cell viability and TNFα resistance. In UMSCC1, depletion of these components significantly reduced clonogenic colony formation (Fig. 3C; representative images in Supplementary Fig. S4A). Interestingly, depletion of PLK1, AURKA, TPR, or NDC80 plus TNFα further significantly reduced colony formation than depletion alone (Fig. 3C; representative images in Supplementary Fig. S4A). Underlying the reduced clonogenicity, flow cytometry analysis revealed that depletion of these five components increased the proportion of cells in the G2/M phase of the cell cycle, as well as increasing sub-G1 DNA, indicative of DNA fragmentation and cell death (Fig. 3D; Supplementary Table S5A, representative images in Supplementary Fig. S4B). The addition of TNFα enhanced the increase in sub-G1 DNA, which reached statistical significance in AURKA- and TPR-depleted cells. To examine the effects on the induction of apoptosis, we performed Annexin V assays by flow cytometry. Depletion of TPR, NUF2, or NDC80 significantly enhanced TNFα-induced apoptosis, whereas PLK1 or AURKA knockdown alone potently induced significant apoptosis, and the increase in apoptotic cells with addition of TNFα approached but did not reach statistical significance (Fig. 3E; representative images in Supplementary Fig. S4C). Supporting the biologic and clinical relevance of these five components in proliferation and mitosis of HNSCC, each of these five genes is expressed at significantly higher levels in HNSCC tumors compared with normal tissue (Supplementary Fig. S5A), especially in HPV^+^ HNSCCs (Supplementary Fig. S5B) in the TCGA dataset. Together, the data above support increased expression of these G2/M kinases and kinetochore components in HNSCC tumors and their functional role in the modulation of NF-κB nuclear localization and activity, as well as promoting proliferation, viability, and TNF resistance in HNSCC cells.

### Inhibition of TTK reduces NF-κB activity in HNSCC cells

**Figure 4:**
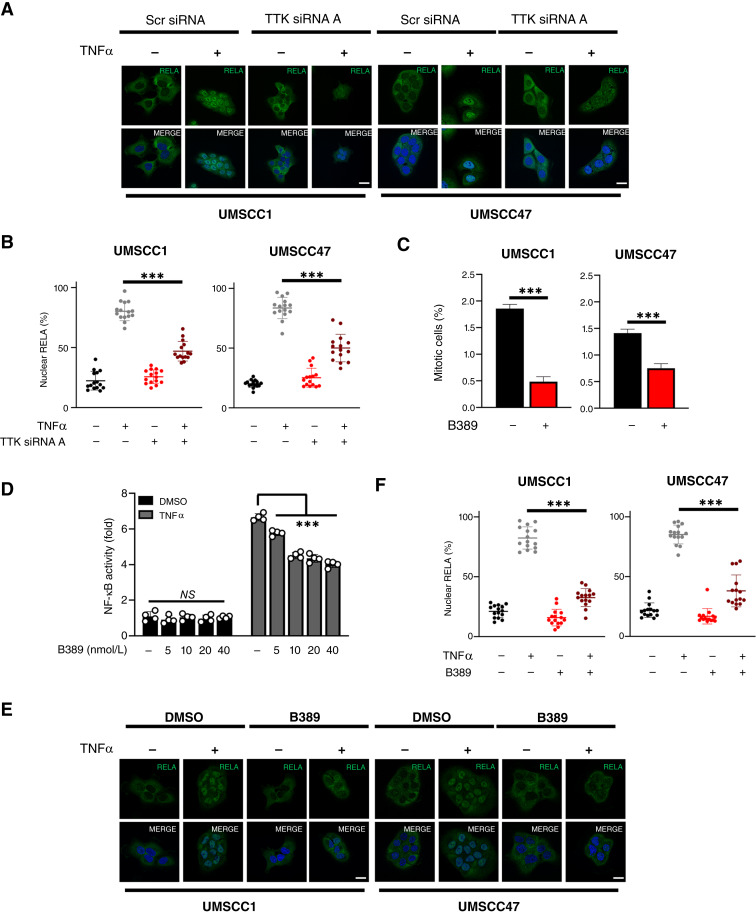
TTK inhibition reduces NF-κB RELA nuclear localization and activity in HNSCC cells. **A,** Representative immunofluorescence images of RELA localization in UMSCC1 and UMSCC47 cells. Cells were transfected with TTK siRNA or control siRNA for 72 hours, with TNFα (20 ng/mL) added for 30 minutes. DAPI was used as a nuclear counterstain. **B,** Quantification of percentage nuclear RELA from **A**. Data represent the percentage nuclear localization of RELA from 15 cells and were analyzed using ImageJ as previously (12). Bars represent means ± SD. **C,** UMSCC1 and UMSCC47 cells were treated with 20 nmol/L BAY1217389 (B389) for 24 hours. Cells were then analyzed for the percentage of phosphorylated histone H3 (Ser10), a marker of mitosis. **D,** NF-κB reporter activity after treatment with increasing doses of B389 in UMSCC1^κB^ cells. Cells were treated with increasing doses of B389 or vehicle control for 24 hours, with TNFα (20 ng/mL) added for the final 16 hours. **E,** Representative immunofluorescence images of RELA localization in UMSCC1 and UMSCC47 cells. Cells were treated with B389 (20  nmol/L) or vehicle control for 6 hours, with TNFα (20 ng/mL) added for 30 minutes. DAPI was used as a nuclear counterstain. **F,** Quantification of percentage nuclear RELA from **E**. Data represent the percentage nuclear localization of RELA from 15 cells and were analyzed using ImageJ as previously (12). All experiments are representative of at least three biological replicates. NS, not significant; ***, *P* < 0.001.

Although WEE1, PLK1, and AURKA kinase inhibitors have been studied in HNSCC and other cancers, small molecule inhibitors targeting other G2/M kinases such as TTK are currently in preclinical or clinical development (36, 37). TTK is a critical kinase that is a key regulator of the mitotic spindle assembly checkpoint (SAC). TTK-dependent SAC activation contributes to the maintenance of genome stability by delaying chromosome segregation until chromosomes are properly attached to the mitotic spindle in metaphase (38). We selected TTK for additional studies for several reasons: (i) TTK knockdown inhibited NF-κB activity in our screens; (ii) TTK has not previously been shown to play a role in TNF sensitization or the NF-κB pathway; (iii) a role for TTK as a potential target in HNSCC has not previously been explored; and (iv) TTK small molecule inhibitors are available and in preclinical trials. To validate the potential role of TTK in NF-κB activation, we first examined the effect of TTK depletion on TTK expression and TNFα-induced NF-κB nuclear translocation. TNFα induced a small increase in TTK expression, whereas two independent TTK siRNAs resulted in reduced basal and TNF-inducible TTK in an HPV^−^ (UMSCC1) and HPV^+^ (UMSCC47) cell line (Supplementary Fig. S6A). TTK depletion also resulted in a significant inhibition of TNFα-induced NF-κB nuclear translocation (Fig. 4A and B). We next confirmed our results using BAY1217389 (hereafter called B389), a highly potent and specific inhibitor of TTK that has undergone phase I clinical testing (39). To assess the functional effect of TTK inhibition, we monitored phosphorylated histone H3 at Ser10, a marker of mitotic cells, which has been extensively used as a marker for TTK activity (40). B389 at a low nanomolar concentration (20 nmol/L) significantly reduced the proportion of mitotic cells after 24 hours of treatment in UMSCC1 and 47 cells (Fig. 4C). Furthermore, this dose was able to reduce TNFα-inducible NF-κB reporter activity, which was decreased in a dose-dependent manner in response to TTK inhibition (Fig. 4D), consistent with TTK siRNA depletion. Functionally, pharmacologic TTK inhibition also significantly reduced TNFα-induced RELA nuclear translocation in both cell lines (Fig. 4E and F). Together, these data confirm that TTK can modulate TNFα-induced NF-κB nuclear translocation and transcriptional activity in HNSCC cells.

### TTK inhibition enhances TNF sensitization in HNSCC cells

**Figure 5:**
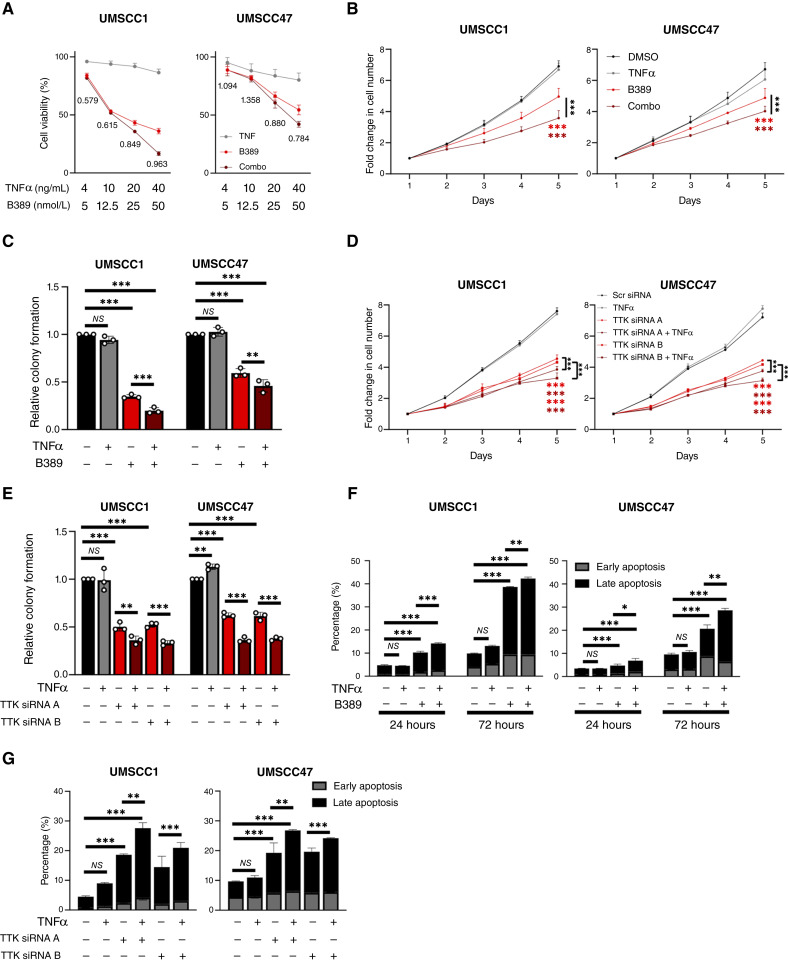
TTK activity promotes TNF resistance in HNSCC cells. **A,** XTT cell viability analysis of UMSCC1 and UMSCC47 cells after treatment with varying doses of B389 and/or TNFα for 48 hours. Values below the combination are CIs as described in the text. **B,** Cell growth analysis of UMSCC1 and UMSCC47 cells after treatment with TNFα (20 ng/mL), B389 (20 nmol/L), or the combination for 24 hours. **C,** Colony formation assay of UMSCC1 and UMSCC47 cells after treatment with TNFα (20 ng/mL), B389 (20 nmol/L), or the combination for 24 hours. **D,** Cell growth analysis of UMSCC1 and UMSCC47 cells after transfection of two specific TTK siRNAs for 72 hours, with or without TNFα addition for the final 24 hours. **E,** Colony formation assay of UMSCC1 and UMSCC47 cells after transfection of two specific TTK siRNAs for 72 hours, with or without TNFα addition for the final 24 hours. **F,** Annexin V analysis of UMSCC1 and UMSCC47 cells after treatment with TNFα (20 ng/mL), B389 (20 nmol/L), or the combination for 24 and 72 hours. **G,** Annexin V analysis of UMSCC1 and UMSCC47 cells after transfection of two specific TTK siRNAs for 72 hours, with or without TNFα addition for the final 24 hours. Bars represent means ± SD. All experiments are representative of at least three biological replicates. NS, not significant; *, *P* < 0.05; **, *P* < 0.01; ***P < 0.001.

We next assessed if TTK activity promotes TNF resistance in HNSCC cells. B389 alone significantly reduced cell viability in a dose-dependent manner; however, the combination of B389 and TNFα further reduced the viability in all cell lines compared with B389 alone (Fig. 5A). To determine if this combination was additive or synergistic, we calculated the CI (21). In UMSCC1 cells, the combination treatment was synergistic at each concentration tested; in UMSCC47, synergism was observed at the higher concentrations (Fig. 5A; CI <1). Correspondingly, B389 significantly reduced cell growth and colony formation at doses that inhibited NF-κB activity, and this was further enhanced in the presence of TNFα (Fig. 5B and C; representative images in Supplementary Fig. S7A). Similar results were obtained upon TTK depletion with two independent siRNAs (Fig. 5D and E; representative images in Supplementary Fig. S7B). Additionally, TTK inhibition with B389 induced increasing apoptosis between early and later time points (24 and 72 hours posttreatment), which was significantly enhanced in combination with TNFα (Fig. 5F; representative images in Supplementary Fig. S7C). Similar results were obtained upon siRNA depletion of TTK (Fig. 5G; representative images in Supplementary Fig. S7D). Together, these data suggest that TTK activity promotes proliferation, survival, and TNF resistance in HNSCC cells.

### TTK inhibition induces mitotic abnormalities and genomic instability in combination with radiation treatment

**Figure 6:**
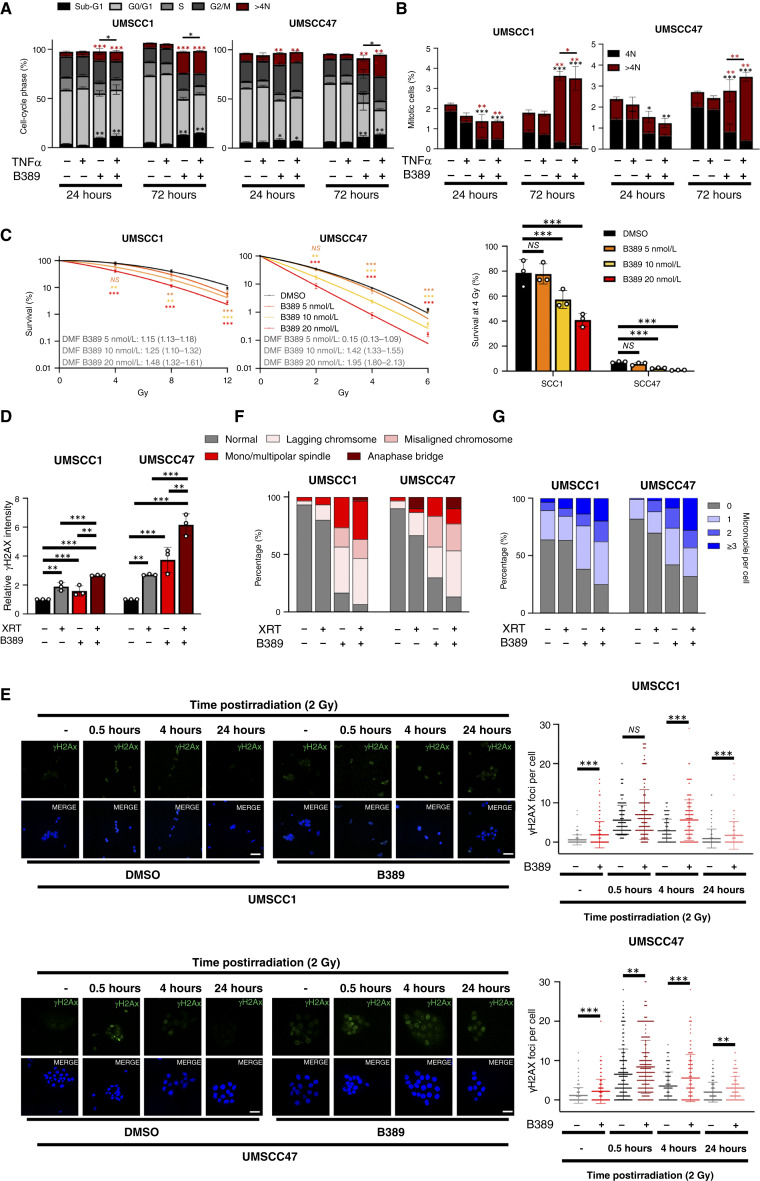
TTK inhibition induces mitotic abnormalities and genome instability in combination with radiation treatment. **A,** Cell-cycle analysis of UMSCC1 and UMSCC47 cells were treated with 20 nmol/L B389 for 24 and 72 hours. Red asterisks indicate a significant difference in the >4N population compared with control; black asterisks indicate a significant difference in the sub-G1 population compared with control. Bars indicate a significant difference in the sub-G1 population between B389 and the combination treatment. Statistics are shown in Supplementary Table S5. **B,** UMSCC1 and UMSCC47 cells were treated with 20 nmol/L B389 for 24 and 72 hours. Cells were then analyzed for the percentage of phosphorylated histone H3 (Ser10), a marker of mitosis, and their DNA content using PI. Red asterisks indicate a significant difference in the >4N population compared with control; black asterisks indicate a significant difference in the sub-G1 population compared with control. Bars indicate a significant difference in the >4N population between B389 and the combination treatment. Statistics are shown in Supplementary Table S5. **C,** left, Clonogenic survival assays of UMSCC1 and UMSCC47 treated with B389 at the indicated doses for 2 hours before irradiation. Cells were then immediately harvested and replated after irradiation. Right, Survival fraction at 4  Gy from each cell line. DMF values were calculated as the vehicle radiation dose for 10% survival divided by radiation dose for 10% survival with the indicated treatment. **D,** UMSCC1 and UMSCC47 cells were treated with B389 (20 nmol/L) for 2 hours before irradiation. Cells were then analyzed for γH2AX expression by flow cytometry after 24 hours. For nonirradiated samples, cells were treated with vehicle or B389 (20  nmol/L) for 24 hours before analysis. Data are presented as the relative MFI compared with the nonirradiated control samples. **E,** left, Representative immunofluorescence images of γH2AX foci in UMSCC1 and UMSCC47 cells after treatment with B389 (20 nmol/L) and/or radiation. Cells were treated with B389 (20 nmol/L) for 2 hours before irradiation. Cells were then analyzed for γH2AX foci by immunofluorescence analysis at the indicated time points. For nonirradiated samples, cells were treated with vehicle or B389 (20 nmol/L) for 24 hours before analysis. DAPI was used as a nuclear counterstain. Scale bar, 10 μmol/L (right). Quantification of γH2AX foci. For each condition, foci from at least 200 cells were counted. **F,** Cells were treated as in **D**. After 24 hours, mitotic cells were analyzed by confocal microscopy for the appearance of mitotic aberrations. Cells were probed for phosphorylated histone H3 (Ser10) and α-tubulin, and DAPI was used as a nuclear counterstain. The percentage of each type of mitotic aberrations is presented from at least 30 individual cells from duplicate experiments. **G,** Cells were treated as in **D**. After 24 hours, cells were analyzed by confocal microscopy for the appearance of micronuclei using DAPI as a nuclear counterstain. The percentage of cells with 0, 1, 2, or ≥3 associated micronuclei is presented at least 200 individual cells from duplicate experiments. Bars represent means ± SD. All experiments are representative of at least three biological replicates, except (**F** and **G**), which are from duplicate experiments. *, *P* < 0.05; **, *P* < 0.01; ***, *P* < 0.001. NS, not significant; MFI, median fluorescence intensity; propidium iodide; DMF, dose modification factor, XRT, X-ray therapy.

TTK plays a critical role in the SAC, and TTK inhibition can induce severe chromosome mis-segregation, polyploidy, and mitotic cell death (36, 38, 40, 41). Consistent with its role at the G2/M checkpoint, TTK inhibition increased the proportion of cells in the G2/M phase of the cell cycle, 24 hours posttreatment (Fig. 6A; Supplementary Table S5B). The proportion of sub-G1 DNA, indicating DNA fragmentation, was also increased upon TTK inhibition, and was enhanced in the presence of TNF, consistent with our previous data. Additionally, TTK inhibition increased the proportion of cells with >4N DNA, indicating the induction of polyploidy. By 72 hours, the proportion of cells with >4N DNA increased further, but this was not further enhanced in combination with TNF treatment (Fig. 6A; representative images in Supplementary Fig. S8A). Polyploidy is often induced because of mitotic abnormalities (42). As we observed an increase in polyploid cells, we looked at the mitotic cell population, as TTK inhibition can lead to mitotic exit and polyploidy (43). As expected, TTK inhibition decreased the percentage of mitotic cells compared with the control at 24 hours (Fig. 6B). However, by 72 hours, the number of mitotic cells increased in both cell lines, and this was only in cells with >4N DNA, consistent with failed mitotic checkpoint in TTK-inhibited cells (Fig. 6B).

TNFα-dependent and radiation-induced cytotoxicity is attenuated by NF-κB activity in many cancers, including HNSCC (6, 44, 45). Furthermore, radiation treatment can induce polyploidy and mitotic defects (46). As TTK inhibition reduced NF-κB activity and sensitized cells to TNFα-induced cytotoxicity, we assessed whether HNSCC cells could also be sensitized to radiation treatment by TTK inhibition. Cells were treated with B389 for 1 hour, followed by exposure to increasing doses of radiation before clonogenic survival was assessed. TTK inhibition enhanced the response to radiation treatment in a dose-dependent manner, with dose modification factor values ranging from 1.15 to 1.48 in UMSCC1 and 1.05 to 1.42 in UMSCC47 cells, respectively (Fig. 6C). Similar radiosensitization occurred upon TTK depletion, with a greater effect observed in HPV^+^ UMSCC47 cells, similar to that observed after B389 treatment (Supplementary Fig. S9A).

To investigate the mechanism of this further, we examined the DNA damage response (DDR) in cells treated with B389 and radiation. In control cells, ɣH2AX expression, a marker for DNA damage (47), was enhanced above control levels in both irradiated cells and in B389-treated cells (Fig. 6D; representative images in Supplementary Fig. S8B); however, combination-treated cells had higher levels of ɣH2AX expression in both cell lines when compared with either treatment alone. To examine the kinetics of DNA damage in TTK-inhibited cells, we performed a time course experiment and looked at ɣH2AX foci resolution. ɣH2AX foci peaked 30 minutes postirradiation and decreased to background levels by 24 hours (Fig. 6E). In contrast, TTK inhibition increased ɣH2AX expression under basal conditions and enhanced and prolonged ɣH2AX expression up to 24 hours postirradiation (Fig. 6E). These data suggest that TTK inhibition induced radiosensitization in HNSCC cells at least in part by impairing DNA damage repair.

Decreased survival upon radiation treatment could possibly be enhanced by the induction of mitotic abnormalities induced upon TTK inhibition. We therefore treated cells with B389 with or without radiation treatment for 24 hours and examined mitotic cells by confocal microscope for signs of mitotic defects. Radiation treatment led to an increase in mitotic defects, including lagging chromosomes and spindle defects (Fig. 6F; example images of mitotic defects are shown in Supplementary Fig. S10A, S10C, and S10D). B389 alone induced significant mitotic defects; combination treatment resulted in >90% of mitotic cells having defects. Similar results were obtained upon TTK depletion (Supplementary Fig. S9B). Finally, as mitotic defects and persistent DNA damage can lead to genomic instability, we looked at the formation of micronuclei, an indicator of genomic instability (48). TTK inhibition alone induced a significant proportion of cells having micronuclei, which was enhanced in combination with radiation treatment (Fig. 6G; example images of micronuclei in UMSCC1 cells are shown in Supplementary Fig. S10B–S10D). Again, similar results were obtained upon TTK depletion (Supplementary Fig. S9C). These data together demonstrate that TTK inhibition radiosensitizes HNSCC cells through the accumulation of mitotic defects, significant DNA damage, and genomic instability.

### TTK expression correlates with survival in HNSCC

**Figure 7:**
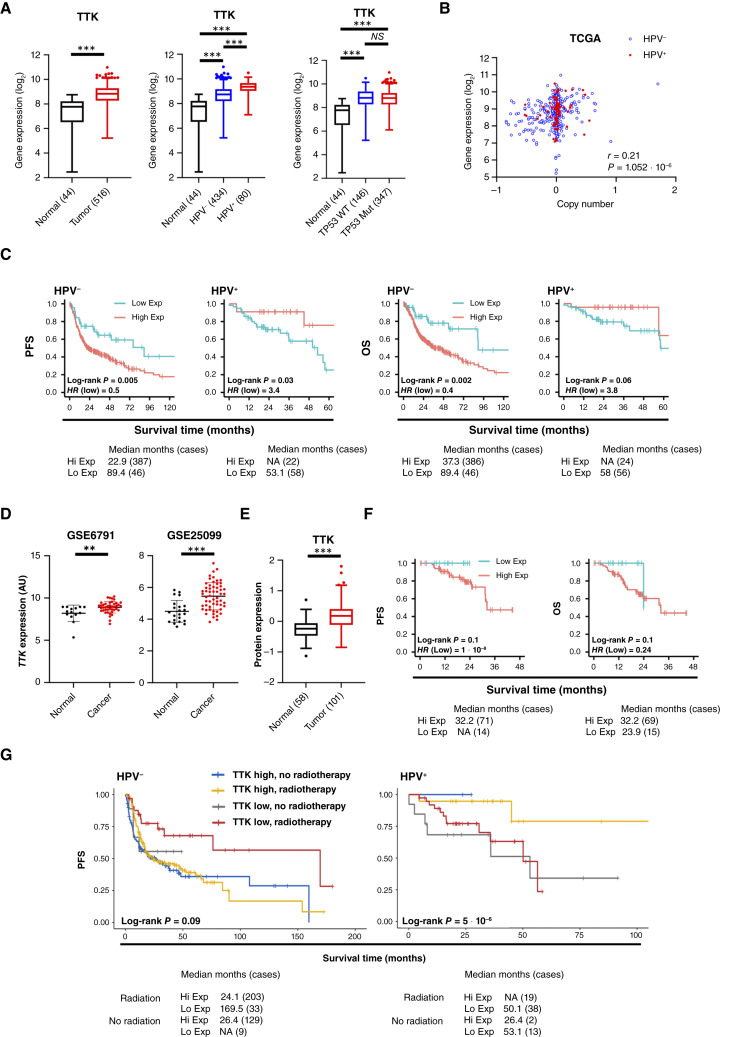
High TTK expression correlates with survival in HNSCC. **A,** Box plot analysis of *TTK* mRNA expression in normal (*n* = 44) and HNSCC (*n* = 516) tissue, in normal (*n* = 44), HPV^−^ HNSCC (*n* = 434) and HPV ^+^ HNSCC (*n* = 80) tissue and in normal (*n* = 44), WT TP53 HNSCCs (*n* = 167), and mtTP53 HNSCCs (*n* = 347) tissue from TCGA HNSCC database. **B,** Correlation of gene copy number with mRNA expression in HNSCC based on HPV status. CNVs were presented on the *X* axis, as one-copy loss (−1), diploid (0), one-copy gain (0.58), and amplification (larger than 1). mRNA expression calculated by RSEM is presented in the *y* axis as the log_2_ scale. Pearson correlation tests were conducted for 508 HNSCC tumor specimens, including 428 HPV^−^ (blue circles) and 80 HPV(+) samples (red triangles). *P* values give the significance of the Pearson correlation between mRNA expression and CNV. *R* values represent Pearson correlation coefficients. **C,** PFS and OS analysis of TCGA HNSCC data based on *TTK* expression, separated by HPV status. Survival data were plotted using the Kaplan–Meier survival curve. Red indicates high expression, and cyan indicates low expression. *P* values were determined using the log-rank test. Plots were truncated at 10 years for HPV^−^ patients and 5 years for HPV^+^ patients, but the analyses were conducted using all data. **D,** Scatter dot plot of *TTK* mRNA expression in normal and HNSCC tissue from the GEO database entries GSE6791 (normal *n* = 14, HNSCC *n* = 42) and GSE25099 (normal *n* = 22, HNSCC *n* = 57). **E,** Box plot analysis of TTK protein expression in normal (*n* = 58) and HNSCC (*n* = 101, all HPV^−^) tissue from the CPTAC dataset. **F,** PFS and OS analysis of CPTAC HNSCC data based on TTK protein expression. Survival data were plotted using the Kaplan–Meier survival curve from 85 HPV^−^ HNSCCs cases that had survival data. Red indicates high expression, and cyan indicates low expression. *P* values were determined using the log-rank test. **G,** PFS analysis of TCGA HNSCC data based on *TTK* expression and if patients were treated with radiotherapy or not, separated by HPV status. Survival data were plotted using the Kaplan–Meier survival curve. Blue indicates high expression with no radiotherapy, gold indicates high expression with radiotherapy, gray indicates low expression with no radiotherapy, and red indicates low expression with radiotherapy. From the TCGA data, 59 HPV^−^ and 8 HPV^+^ cases had no radiotherapy record, so they were excluded from the analysis. *P* values were determined using the log-rank test. *, *P* < 0.05; **, *P* < 0.01; ***, *P* < 0.001 (Student *t* test). NS, not significant; CNV, copy number variations; GEO, Gene Expression Omnibus; WT, wildtype.

To assess the potential clinical relevance of TTK in HNSCC, we assessed the expression levels using the TCGA database. Analysis of the TCGA HNSCC cohort demonstrated that *TTK* mRNA was expressed at significantly higher levels in HNSCC tumors than in normal tissue (Fig. 7A). Furthermore, *TTK* mRNA was expressed at higher levels in HPV^+^ tumors. *TTK* mRNA expression was significantly correlated with the gene copy number (Fig. 7B). We next investigated the relationship between *TTK* mRNA expression and patient survival. High *TTK* mRNA expression was significantly associated with worse PFS and OS in HPV^−^ HNSCCs (Fig. 7C). However, in HPV^+^ HNSCCs, *TTK* mRNA expression correlated with a better prognosis (significant for PFS but not OS). Similar mRNA expression trends were observed in tumors from two other cohorts of patients with HNSCC (Fig. 7D). In confirmation of the transcriptomic results, we observed increased TTK protein expression in cancer tissue from the CPTAC database when compared with normal oral tissue (Fig. 7E; ref. 49). In this dataset, high expression of TTK protein expression also trended toward worse PFS and OS, although this did not reach statistical significance, potentially due to the smaller sample size compared with the TCGA dataset (Fig. 7F). Finally, to ascertain if there was a prognostic association between TTK, radiosensitivity, and tumor stage in the TCGA HPV^−^ and HPV^+^ HNSCC cohorts, we performed a multivariate analysis (Fig. 7G). In HPV^−^ tumors, the *P* value for TTK expression remains significant (0.013), with a HR = 1.99, indicating a strong relationship between TTK expression and decreased survival. Interestingly, the *P* value for adding radiotherapy is 0.014, with a HR = 0.67, indicating a strong relationship between adding radiotherapy and decreased risk of death. Compared with surgery only, surgery and radiation reduces the hazard by a factor of 0.57, or 43%. As expected, the *P* value for stage is also significant (0.003), with a HR = 1.83, indicating a very strong relationship between advanced stage and decreased survival. For HPV^+^ patients, the association of TTK expression was borderline significant (*P* = 0.058) with a HR = 0.24, suggesting higher TTK expression is associated with decreased risk of death. When controlling for the other variables, the difference with addition of radiation (HR = 0.78; *P* = 0.62) or higher stage (HR 1.72; *P* = 0.60) does not reach significance. Taken together, these data suggest that the expression of several mitotic kinases and kinetochore components is upregulated in HNSCC, particularly in the HPV^+^ subtype. Furthermore, we demonstrate that TTK has a differential relationship with survival dependent on HPV status, advanced tumor stage, and addition of radiotherapy.

## Discussion

Here, overlapping kinome, DG, WG, and validation siRNA screens reproducibly identified multiple candidate targets that significantly modulate TNFα-induced NF-κB activity and/or cell viability of an HNSCC reporter cell line. Pathway analyses revealed significant enrichment for components of canonical NF-κB signaling, as well as protein ubiquitination and other signal pathways previously associated with NF-κB activity, and unexpectedly, multiple candidates linked to the kinetochore and G2/M cell-cycle checkpoint. A validation screen and further functional studies with independent siRNAs or pharmacologic inhibitors of G2/M kinases and structural components demonstrated reduced TNFα-induced NF-κB reporter activity and nuclear localization of RELA, suggesting these effects may be linked to RELA nuclear translocation, an essential step in activation of the canonical NF-κB pathway (50, 51). Detailed investigation of the mitotic kinase TTK demonstrated similar effects on NF-κB RELA translocation and activity, as well as cell viability, cell death, and TNFα resistance in both HPV^−^ and HPV^+^ HNSCC cells. Furthermore, TTK inhibition significantly enhanced radiation genotoxicity, suggesting that TTK could be a potential therapeutic target in HNSCC.

IKK/NF-κB signaling is a critical pathway that is aberrantly activated in many cancers including HNSCC, promoting expression of mediators of cell proliferation, survival, and therapeutic resistance (4–6, 44). Activation of this pathway promotes resistance to the effects of TNFα by inducing transcription of antiapoptotic genes, countering its role as an activator of cell death pathways (4–6, 44). Furthermore, NF-κB promotes resistance to radio-, chemo-, and immune checkpoint therapy, which are often used in the treatment of HNSCC. These therapies also result in significant DNA damage and genotoxic stress that can induce activation of cell-cycle checkpoint pathways that function to arrest the cell cycle at G2, allowing DNA repair before cells with lethal DNA damage undergo mitosis. G2/M cell-cycle control is thus a critical mechanism to maintain genome integrity, cell division, and viability (52). The expression or function of cell-cycle components is often deregulated in cancer cells and therefore represent potential therapeutic targets.

Recently, we demonstrated that one of the targets identified in our RNAi screening, the G2/M checkpoint kinase WEE1, promotes TNFα-induced co-activation of canonical IKKα/β, nuclear translocation of RELA, and NF-κB transactivation, as well as TNFα-induced phosphorylation of known WEE1 target CDC2, identifying a novel role of TNFα in co-activation of these kinases mediating G2/M cell-cycle arrest and of NF-κB prosurvival signaling in HNSCC (5). Here, we present the wider result from our RNAi screening and functional studies, indicating that knockdown of other G2/M checkpoint kinases (PLK1, AURKA, TPR, and TTK), and even structural kinetochore components (NUF2 and NDC80) or microtubule subunit tubulin (TUBA1B), can also inhibit TNFα-induced NF-κB reporter gene activity, RELA nuclear translocation, and cell viability in HNSCC. Our additional functional studies, indicating the role of several of these G2/M kinases and structural kinetochore components in TNFα-induced nuclear localization of RELA, are consistent with prior studies of individual G2/M targets, in which inhibition of PLK1 (30, 35), AURKA (31, 33, 34), microtubule assembly (29, 32), and other kinases that disrupt the G2/M checkpoint (43) also affect the activation of IKK/NF-κB signaling via multiple mechanisms (37).

Previous screening studies have suggested that G2/M components are deregulated and potentially important in the pathogenesis and therapeutic targeting of HNSCC and other cancers (53, 54). HNSCCs are genomically unstable, and this may make them especially vulnerable to therapeutics that together target NF-κB–induced antiapoptotic mechanisms and the G2/M checkpoint, which is essential in HNSCC due to loss of the G1 checkpoint by mutation or HPV-mediated degradation of TP53. The activity of individual PLK1, AURKA, and WEE1 inhibitors observed in both HPV^+^ and HPV^−^ HNSCCs, both alone and in combination with chemoradiation, has been primarily attributed to cell-cycle arrest and aberrant mitoses (53, 55–57). Furthermore, several large-scale screens have identified the G2/M checkpoint and associated genes as critical in cell viability alone or in combination with chemotherapy in HNSCC (53, 54, 58). However, the link shown between WEE1 (5) and herein between TTK and multiple components of the G2/M checkpoint, with NF-κB translocation and activity, and TNF- and radioresistance, has not been previously established.

Here, we chose to focus our experiments on TTK, a critical kinase that is a key regulator of the mitotic SAC. TTK-dependent SAC activation contributes to the maintenance of genome stability by delaying chromosome segregation until chromosomes are properly attached to the mitotic spindle in metaphase (38). Once this is achieved, TTK is inactivated, and cells progress to anaphase. As a result, inhibition of TTK leads to the inactivation of the SAC and results in premature chromosome segregation, leading to aneuploidy, genomic instability, and cell death (40, 41, 43). Several studies have identified TTK and its effects on G2/M as an attractive target in cancer, including in HNSCC (53, 54, 58). As such, several small molecule inhibitors have been developed (59).

Our data demonstrate that HNSCC cells are highly sensitive to TTK inhibition with low nanomolar concentrations of BAY1217389, a potent TTK inhibitor currently in early phase I studies in combination with paclitaxel (39). Treatment with TTK inhibitor alone (or TTK depletion) resulted in a significant decrease in proliferation, accumulation of cells in G2/M of the cell cycle, and subsequently high levels of aneuploidy and cell death. This is likely due to the induction of significant DNA damage, mitotic aberrations, and the appearance of micronuclei, a marker of genomic instability. However, our data demonstrate both that TTK inhibition alone results in unrepaired DNA damage foci, 24 hours posttreatment, and induces DNA damage itself, as well as in combination with radiation. This suggests that TTK may play a role in DNA damage repair, but further studies are required to directly assess this. Interestingly, the effects of TTK inhibition were further enhanced with the addition of TNFα, suggesting that TTK activity contributes to TNF resistance in HNSCC cells. Additionally, TTK inhibition sensitized HNSCC cells to the effects of radiation, resulting in further genomic instability and a decrease in cell survival. Most likely, this is due in large part to the increase in cells in the G2/M stage of the cell cycle, in which cells have been shown to be more radiosensitive (60, 61). As TTK inhibition alone leads to significant DNA damage, combination with radiotherapy may also lead to the DDR being overwhelmed, resulting in a failure to repair DNA damage. Together, these data suggest that targeting TTK may be beneficial in enhancing sensitivity of HNSCC to radiotherapy.

Our data also demonstrated that TTK inhibition decreased the number of mitotic cells at 24 hours but increased the number of mitotic cells at 72 hours, specifically those with >4N DNA, consistent with a failed mitotic checkpoint. This data suggest that TTK inhibitors are primarily effective in highly proliferative cells; alternatively, it may also be related to the time required for most cells at different phases of the cell cycle to progress and cumulatively undergo sufficient damage in G2/M (and cells containing >4N) and subsequent cell death. Therefore, further clinical studies of the pharmacokinetics and pharmacodynamics of TTK inhibitors in tumors could have clinical implications for the scheduling of concurrent or sequential treatment combinations.

Although both HPV^−^ and HPV^+^ HNSCC cells exhibited sensitivity to TTK inhibition and TNFα, patients with HNSCC that received standard surgery or surgery plus radiation treatment in TCGA displayed a differential survival outcome based on TTK expression and HPV status. In multivariate analysis, in HPV^−^ HNSCCs, high TTK expression correlated with worse survival, whereas the outcome was improved with addition of radiation and surgery, consistent with the potential importance of TTK in radiation sensitization. The inverse relationship between TTK expression and survival in HPV^+^ HNSCCs approached but did not quite reach significance, possibly due to smaller sample size. This differing relationship between TTK and survival by HPV status is similar to what we observed with WEE1, in which high expression in HPV^−^ HNSCCs was also associated with previously defined clusters lacking immune signatures, which would also be predicted to respond poorly to standard treatment or immune checkpoint blockade (5, 62). In contrast, HPV^+^ HNSCCs with high WEE1 expression and better survival were observed to associate with copy number alterations/mutations in TRAF3 and/or CYLD, suppressors of NF-κB activation, whose loss was previously associated with increased NF-κB–related inflammatory signatures and better prognosis (63, 64). It would be of interest to see if TTK expression is similarly linked to immune or other gene signatures and if this could potentially explain the differences in survival of patients with standard therapy alone, as we previously observed for expression of WEE1.

TCGA data indicate that the expression of the six G2/M targets studied here is higher in HPV^+^ HNSCC s when compared with HPV^−^ HNSCCs. The reasons behind this are unclear; however, it has been previously demonstrated that HPV^+^ HNSCCs have higher expression of mitotic and DNA damage genes (5, 65, 66). Furthermore, the HPV oncogenes E6 and E7 induce chronic oncogene–induced replication stress and dysregulate the DDR, which make them more susceptible to drugs that target replication stress and homologous recombination repair (67, 68). In line with this, several studies have demonstrated increased sensitivity to TTK inhibition in tumors with high levels of genomic instability (40, 43, 69, 70).

Taken together, our experimental and bioinformatic analyses suggest that several G2/M checkpoint and kinetochore components play a key role in regulating prosurvival NF-κB activity and promoting TNF resistance in HNSCC. Furthermore, our study highlights the mitotic kinase TTK as a potential therapeutic target in HNSCC, of potentially high significance in patients with HPV^−^ tumors and receiving radiotherapy. Because of the potential role of TTK in TNF-induced NF-κB prosurvival, subsequent preclinical and clinical studies are warranted to identify a possible role in combination with chemo-, radio-, or immune therapy.

## Supplementary Material

Figure S1 Figure S1 and figure legend

Figure S2 Figure S2 and figure legend

Figure S3 Figure S3 and figure legend

Figure S4 Figure S4 and figure legend

Figure S5 Figure S5 and figure legend

Figure S6 Figure S6 and figure legend

Figure S7 Figure S7 and figure legend

Figure S8 Figure S8 and figure legend

Figure S9 Figure S9 and figure legend

Figure S10 Figure S10 and figure legend

Table S1-5 legends Supplementary table legends

Table S1 Table S1

Table S2 Table S2

Table S3 Table S3

Table S4 Table S4

Table S5 Table S5

Supplementary Methods Supplementary Methods
