# Supplementary material for: Functional RNAi Screening Identifies G2/M and Kinetochore Components as Modulators of TNFα/NF-κB Prosurvival Signaling in Head and Neck Squamous Cell Carcinoma
Source: Cancer Res Commun. 2024 Nov 7;4(11):2903–18. doi: 10.1158/2767-9764.CRC-24-0274 (PMC11541648; doi:10.1158/2767-9764.CRC-24-0274)
Supplement: Figure S1 — and figure legend [file crc-24-0274_figure_s1_suppsf1.pdf]

Supplementary Figures

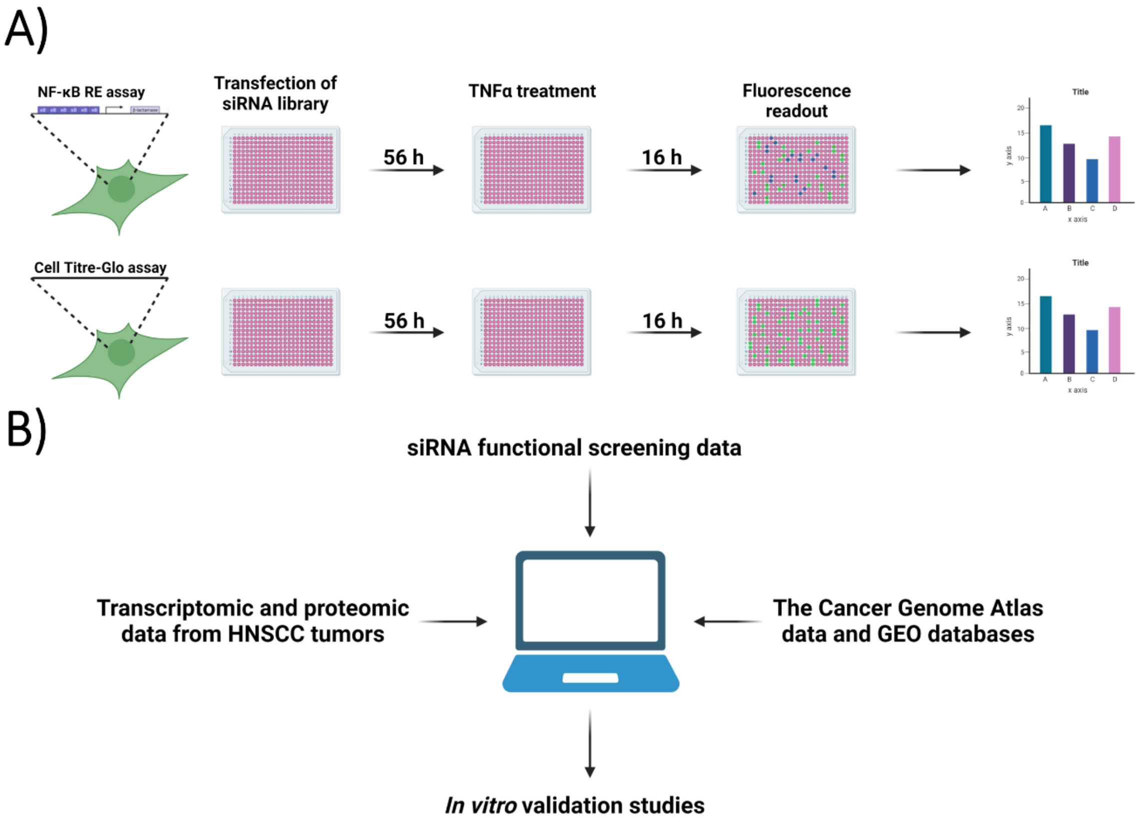

Supplementary Figure 1. Schematic for RNAi screening platform and project pipeline.

A) Schematic for the NF-κB reporter assay in UMSCC1$^{\kappa B}$ cells and the subsequent RNAi screens for NF-κB activity and cell viability. **B)** Integrated approach used in the study.
