# Supplementary material for: Functional RNAi Screening Identifies G2/M and Kinetochore Components as Modulators of TNFα/NF-κB Prosurvival Signaling in Head and Neck Squamous Cell Carcinoma
Source: Cancer Res Commun. 2024 Nov 7;4(11):2903–18. doi: 10.1158/2767-9764.CRC-24-0274 (PMC11541648; doi:10.1158/2767-9764.CRC-24-0274)
Supplement: Figure S2 — and figure legend [file crc-24-0274_figure_s2_suppsf2.pdf]

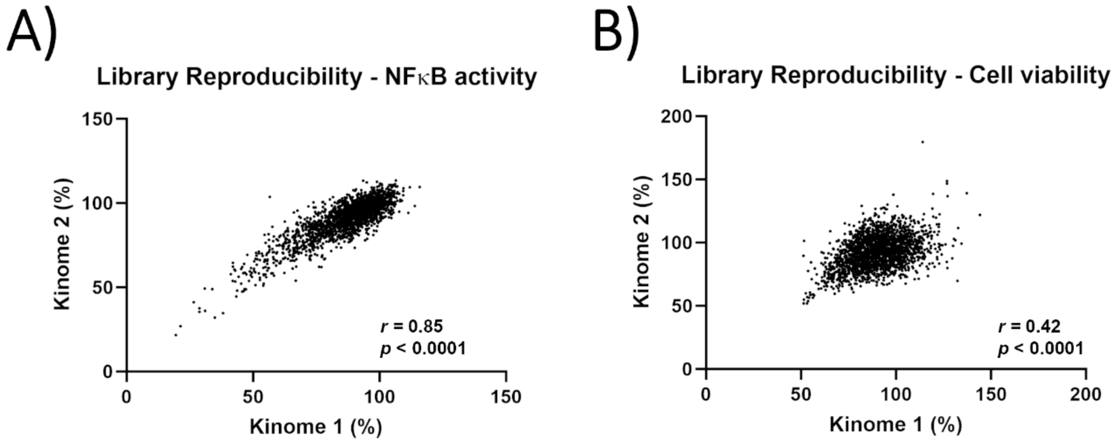

Supplementary Figure 2. RNAi screens demonstrate high levels of reproducibility. A) Correlation between Kinome 1 and Kinome 2 RNAi screen for NF-κB activity. **B)** Correlation between Kinome 1 and Kinome 2 RNAi screen for cell viability.
