# Supplementary material for: Functional RNAi Screening Identifies G2/M and Kinetochore Components as Modulators of TNFα/NF-κB Prosurvival Signaling in Head and Neck Squamous Cell Carcinoma
Source: Cancer Res Commun. 2024 Nov 7;4(11):2903–18. doi: 10.1158/2767-9764.CRC-24-0274 (PMC11541648; doi:10.1158/2767-9764.CRC-24-0274)
Supplement: Figure S3 — and figure legend [file crc-24-0274_figure_s3_suppsf3.pdf]

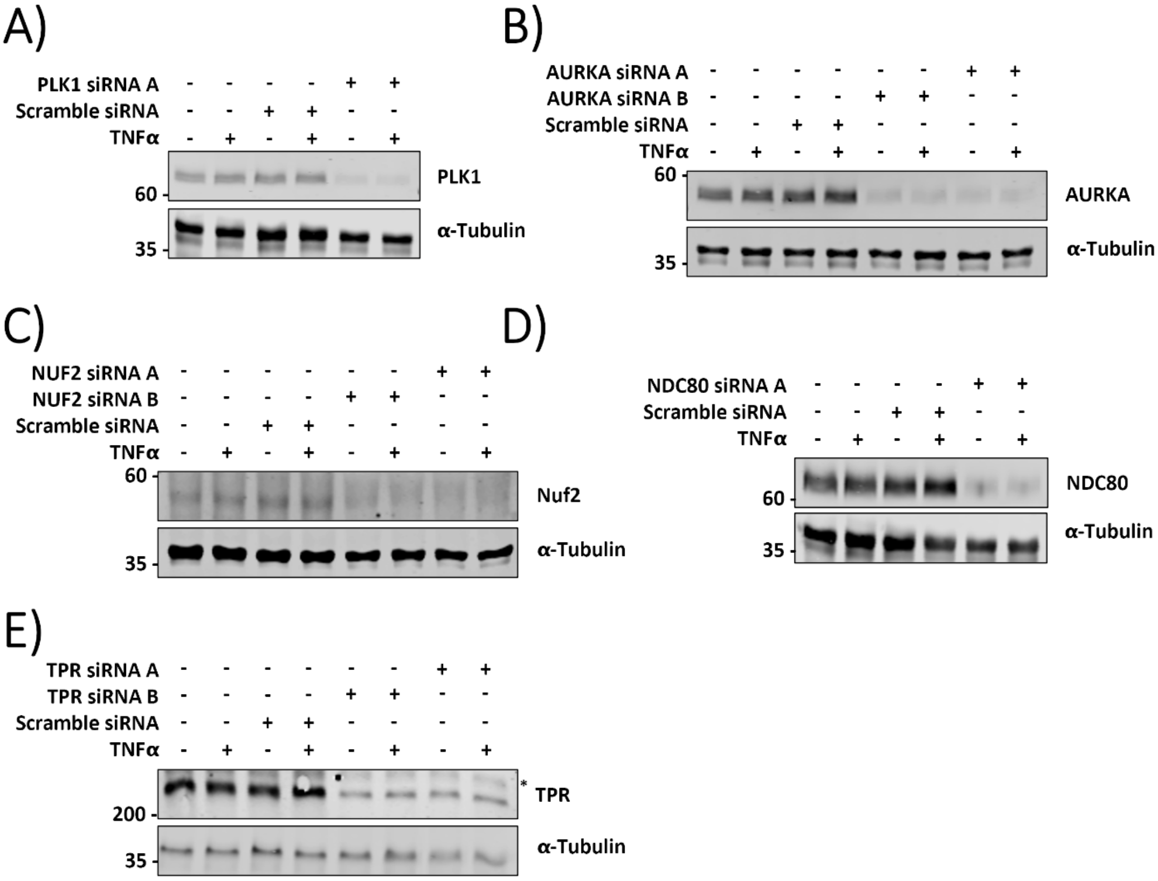

Supplementary Figure 3. Depletion of G2/M kinases and kinetochore proteins in HNSCC cells. A) Representative western blot of UMSCC1 cells after transfection with a specific PLK1 siRNA for 72 h and analysed for PLK1 expression. α-tubulin was used as the loading control. **B)** Representative western blot of UMSCC1 cells after transfection with two specific AURKA siRNA for 72 h and analysed for AURKA expression α-tubulin was used as the loading control. **C)** Representative western blot of UMSCC1 cells after transfection with two specific NUF2 siRNA for 72 h and analysed for NUF2 expression. α-tubulin was used as the loading control. **D)** Representative western blot of UMSCC1 cells after transfection with a specific NDC80 siRNA for 72 h and analysed for NDC80 expression. α-tubulin was used as the loading control. **E)** Representative western blot of UMSCC1 cells after transfection with two specific TPR siRNA for 72 h and analysed for TPR expression. α-tubulin was used as the loading control.
