# Supplementary material for: Functional RNAi Screening Identifies G2/M and Kinetochore Components as Modulators of TNFα/NF-κB Prosurvival Signaling in Head and Neck Squamous Cell Carcinoma
Source: Cancer Res Commun. 2024 Nov 7;4(11):2903–18. doi: 10.1158/2767-9764.CRC-24-0274 (PMC11541648; doi:10.1158/2767-9764.CRC-24-0274)
Supplement: Figure S5 — and figure legend [file crc-24-0274_figure_s5_suppsf5.pdf]

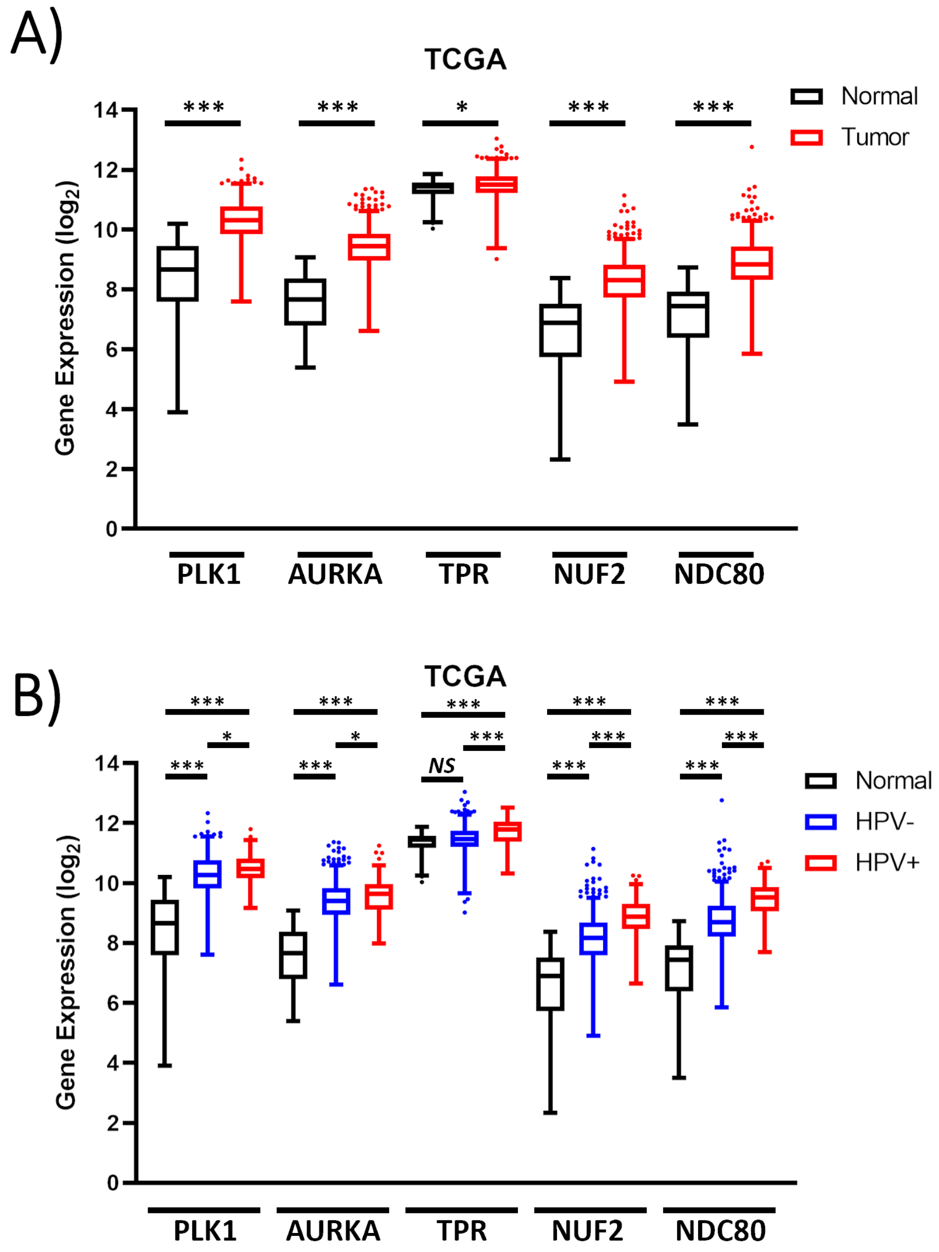

Supplementary Figure 5. Expression of G2/M kinetochore components in TCGA HNSCC. **A)** Box plot analysis of *PLK1, AURKA, TPR, NUF2* and *NDC80* mRNA expression in normal (*n* = 44) and HNSCC (*n* = 516) tissue from TCGA HNSCC database. **B)** Box plot analysis of *PLK1, AURKA, TPR, NUF2* and *NDC80* mRNA expression in normal (*n* = 44), HPV- HNSCCs (*n* = 434) and HPV+ HNSCCs (*n* = 80) tissue from TCGA HNSCC database.
