# Supplementary material for: Functional RNAi Screening Identifies G2/M and Kinetochore Components as Modulators of TNFα/NF-κB Prosurvival Signaling in Head and Neck Squamous Cell Carcinoma
Source: Cancer Res Commun. 2024 Nov 7;4(11):2903–18. doi: 10.1158/2767-9764.CRC-24-0274 (PMC11541648; doi:10.1158/2767-9764.CRC-24-0274)
Supplement: Figure S6 — and figure legend [file crc-24-0274_figure_s6_suppsf6.pdf]

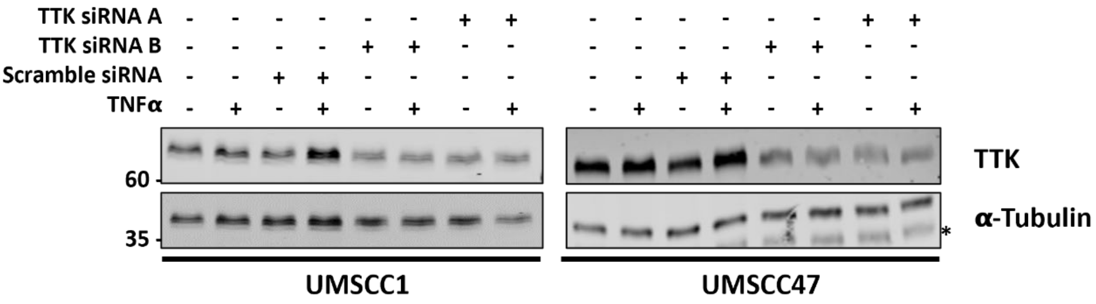


**Supplementary Figure 6. Depletion of TTK in HNSCC cells. A)** Representative western blot of UMSCC1 and UMSCC47 cells after transfection with two specific TTK siRNA for 72 h and analysed for TTK expression. α-tubulin was used as the loading control.
