# Supplementary material for: Functional RNAi Screening Identifies G2/M and Kinetochore Components as Modulators of TNFα/NF-κB Prosurvival Signaling in Head and Neck Squamous Cell Carcinoma
Source: Cancer Res Commun. 2024 Nov 7;4(11):2903–18. doi: 10.1158/2767-9764.CRC-24-0274 (PMC11541648; doi:10.1158/2767-9764.CRC-24-0274)
Supplement: Figure S7 — and figure legend [file crc-24-0274_figure_s7_suppsf7.pdf]

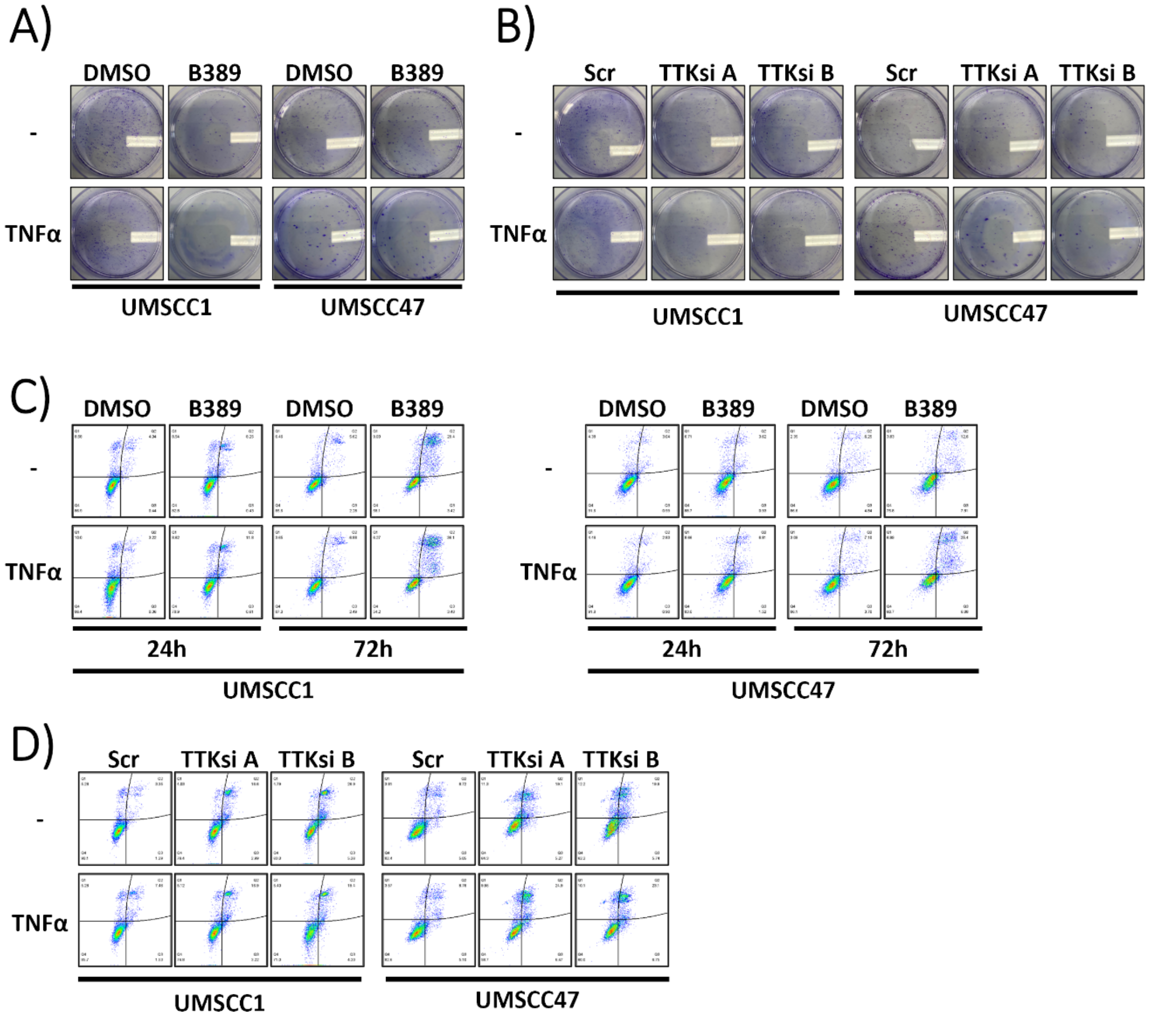

Supplementary Figure 7. Representative images from Figure 5. A) Representative images of colony formation assays in Figure 5C. **B)** Representative images of colony formation assays in Figure 5E. **C)** Representative images of Annexin V assay in Figure 5F. **D)** Representative images of Annexin V assay in Figure 5G.
