# Supplementary material for: Functional RNAi Screening Identifies G2/M and Kinetochore Components as Modulators of TNFα/NF-κB Prosurvival Signaling in Head and Neck Squamous Cell Carcinoma
Source: Cancer Res Commun. 2024 Nov 7;4(11):2903–18. doi: 10.1158/2767-9764.CRC-24-0274 (PMC11541648; doi:10.1158/2767-9764.CRC-24-0274)
Supplement: Figure S8 — and figure legend [file crc-24-0274_figure_s8_suppsf8.pdf]

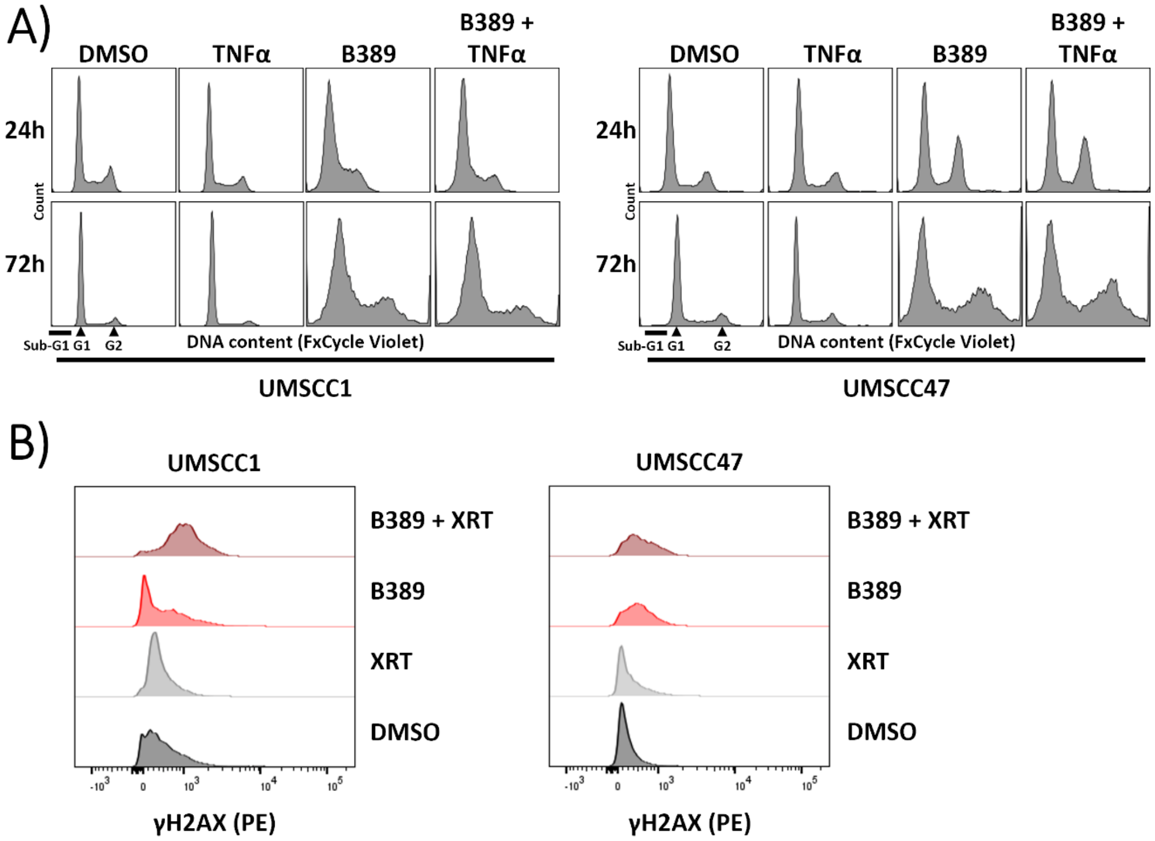

Supplementary Figure 8. Representative images from Figure 6. A) Representative images of cell cycle analysis in Figure 6A. **B)** Representative images of γH2AX intensity in Figure 6D.
