# Supplementary material for: Functional RNAi Screening Identifies G2/M and Kinetochore Components as Modulators of TNFα/NF-κB Prosurvival Signaling in Head and Neck Squamous Cell Carcinoma
Source: Cancer Res Commun. 2024 Nov 7;4(11):2903–18. doi: 10.1158/2767-9764.CRC-24-0274 (PMC11541648; doi:10.1158/2767-9764.CRC-24-0274)
Supplement: Figure S9 — and figure legend [file crc-24-0274_figure_s9_suppsf9.pdf]

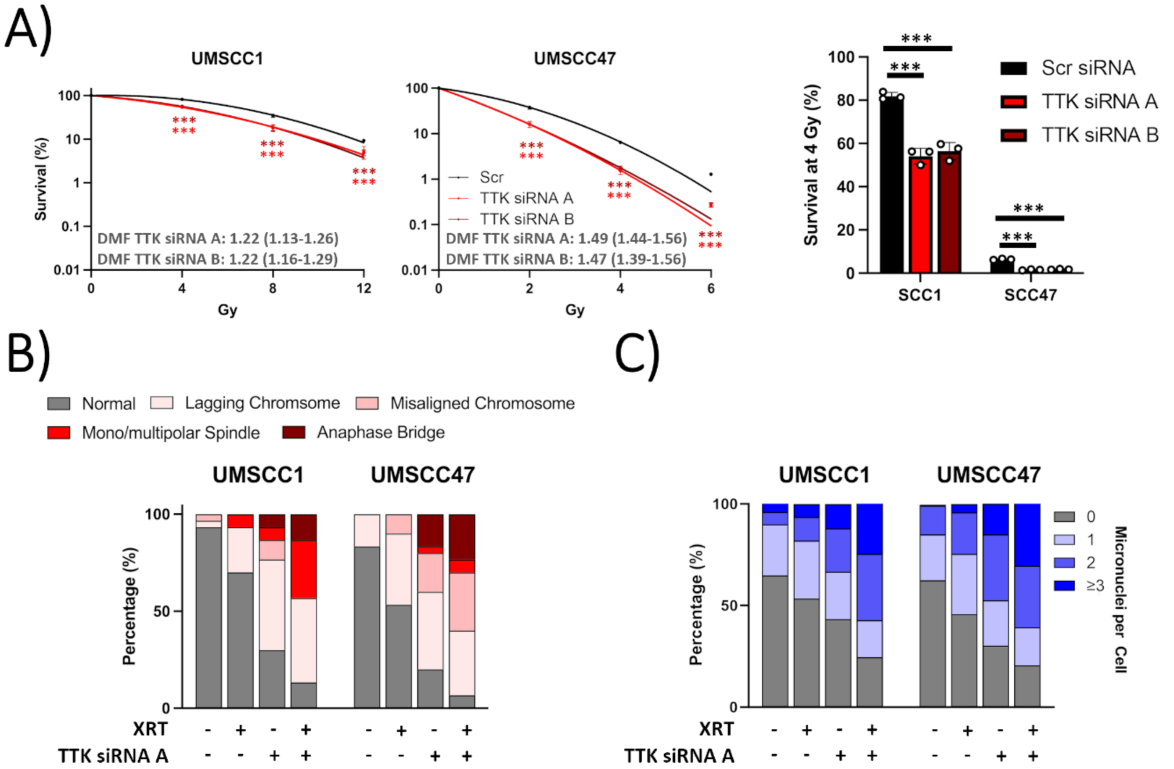

Supplementary Figure 9. TTK depletion sensitizes HNSCC cells to radiation treatment and induces mitotic abnormalities and genome instability. A) (left) Clonogenic survival assays of UMSCC1 and UMSCC47 UMSCC1 and UMSCC47 cells transfected with two specific TTK siRNAs for 72 h before irradiation. Cells were then immediately harvested and replated after irradiation. (right) Survival fraction at 4 Gy from each cell line. DMF values were calculated as the vehicle radiation dose for 10% survival divided by radiation dose for 10% survival with the indicated treatment. **B)** Cells were transfected with two specific TTK siRNAs for 48h. Cells were then irradiated and after 24 h, mitotic cells were analysed by confocal microscopy for the appearance of mitotic aberrations. Cells were probed for phosphorylated Histone H3 (Ser10), α-tubulin and DAPI was used as a nuclear counterstain. The percentage of each type of mitotic aberrations is presented from at least 30 individual cells from duplicate experiments. **C)** Cells were treated as in **B).** After 24 h, cells were analysed by confocal microscopy for the appearance of micronuclei using DAPI as a nuclear counterstain. The percentage of cells with 0, 1, 2 or ≥3 associated micronuclei is presented at least 200 individual cells from duplicate experiments. Bars represent the means ± standard deviation. All experiments are representative of at least three biological replicates, except **B)** and **C)**, which are from duplicate experiments. NS – not significant; *p <0.05, **p < 0.01, ***p < 0.001.
