# Supplementary material for: Functional RNAi Screening Identifies G2/M and Kinetochore Components as Modulators of TNFα/NF-κB Prosurvival Signaling in Head and Neck Squamous Cell Carcinoma
Source: Cancer Res Commun. 2024 Nov 7;4(11):2903–18. doi: 10.1158/2767-9764.CRC-24-0274 (PMC11541648; doi:10.1158/2767-9764.CRC-24-0274)
Supplement: Figure S10 — and figure legend [file crc-24-0274_figure_s10_suppsf10.pdf]

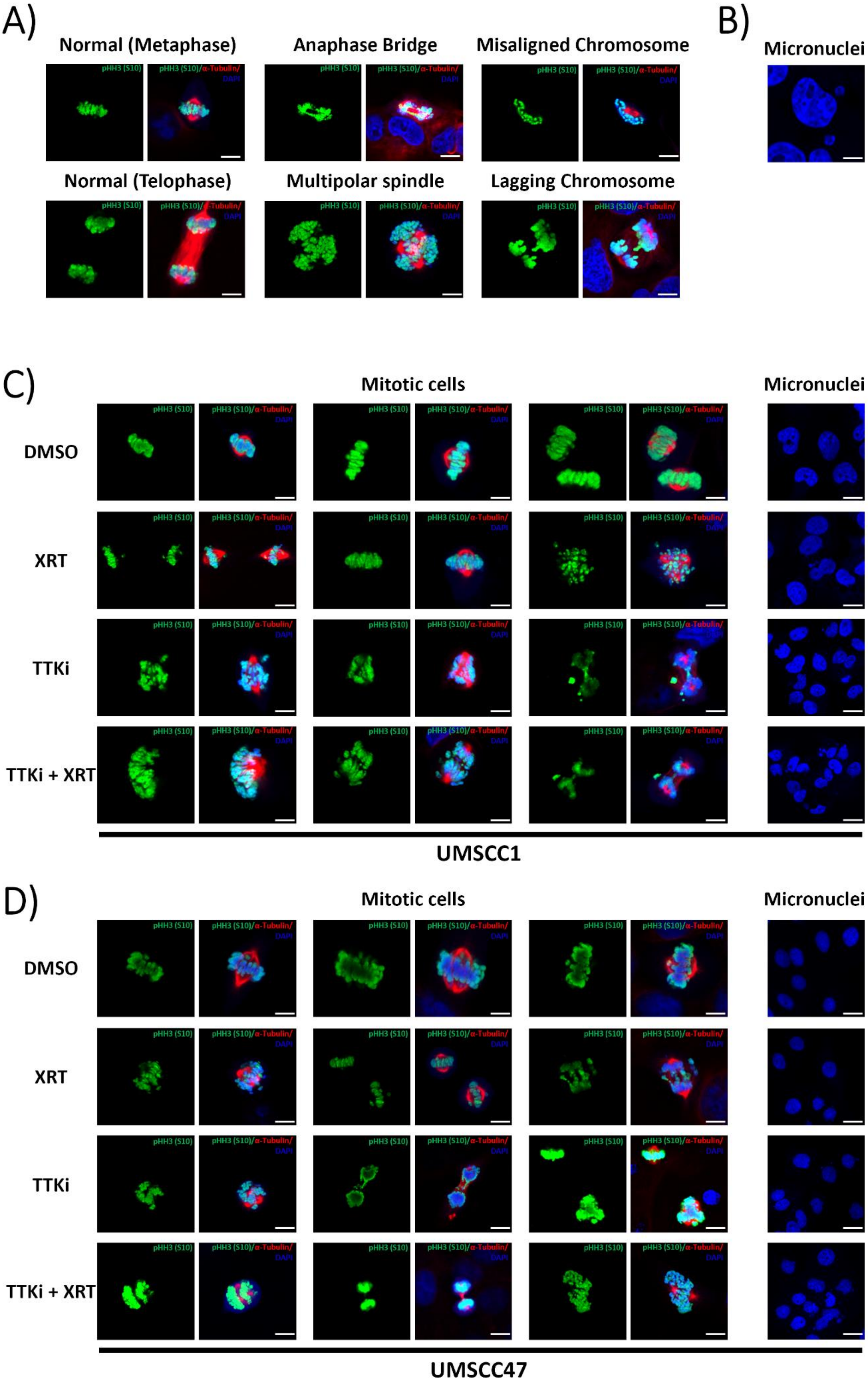
A)
Normal (Metaphase)
Anaphase Bridge
Misaligned Chromosome
Normal (Telophase)
Multipolar spindle
Lagging Chromosome
pHH3 (S10)
pHH3 (S10)/α-Tubulin/DAPI
B)
Micronuclei
C)
Mitotic cells
Micronuclei
DMSO
XRT
TTKi
TTKi + XRT
UMSCC1
D)
Mitotic cells
Micronuclei
DMSO
XRT
TTKi
TTKi + XRT
UMSCC47

Supplementary Figure 10. Examples of normal mitotic cells, aberrant mitoses, and micronuclei. **A)** Representative immunofluorescence images of normal mitotic cells (metaphase and telophase, as well as several aberrant mitoses (anaphase bridges, misaligned chromosomes, multipolar spindles and lagging chromosomes). Cells were probed for phosphorylated Histone H3 (Ser10), α-tubulin and DAPI was used as a nuclear counterstain. **B)** Representative immunofluorescence images of micronuclei. DAPI was used as a nuclear counterstain. **C)** Representative immunofluorescence images of mitotic cells (left) and nuclei (right) in UMSCC1 cells after treatment with radiation (XRT), B389 or combination treatment. Cells were probed for phosphorylated Histone H3 (Ser10), α-tubulin and DAPI was used as a nuclear counterstain. **D)** Representative immunofluorescence images of mitotic cells (left) and nuclei (right) in UMSCC47 cells after treatment with radiation (XRT), B389 or combination treatment. Cells were probed for phosphorylated Histone H3 (Ser10), α-tubulin and DAPI was used as a nuclear counterstain.
