# Supplementary material for: Functional RNAi Screening Identifies G2/M and Kinetochore Components as Modulators of TNFα/NF-κB Prosurvival Signaling in Head and Neck Squamous Cell Carcinoma
Source: Cancer Res Commun. 2024 Nov 7;4(11):2903–18. doi: 10.1158/2767-9764.CRC-24-0274 (PMC11541648; doi:10.1158/2767-9764.CRC-24-0274)
Supplement: Table S1-5 legends — Supplementary table legends [file crc-24-0274_table_s1-5_legends_suppstl.docx]

**Supplementary Tables**
**Supplementary Table 1.** Individual siRNA data from **(A)** both kinome 1 and 2 and **(B)** druggable genome screens. Data includes both the NF-κB reporter activity (%), cell viability (%) of the siRNA Negative control, Median absolute deviation (MAD) ranking, Redundant siRNA Analysis (RSA) LogP ranking, and p value and q value for each siRNA used.  **Supplementary Table 2.** Individual siRNA data from the whole genome screen screens. Data includes **(A)** the NF-κB reporter activity (%), cell viability (%) of the siRNA Negative Control, Median absolute deviation (MAD) score, LogP values for siRNAs and **(B)** for each gene after common seed analysis (CSA) correction.  **Supplementary Table 3.** Data from validation studies of selected hits of potential interest from the whole genome screen screen and IPA**. A)** Data shows individual siRNA data from both primary WG and secondary validation screens showing NF-κB reporter activity (%), Z scores and seed corrected Z scores. **B)** Gene level data showing NF-κB reporter activity (%), Z scores and seed corrected Z scores.  **Supplementary Table 4.** Ingenuity Pathway Analysis (IPA) on the 769 genes with significance of LogP< -2 following CSA of the whole genome screen (Table S2B). **Supplementary Table 5.** Statistical analysis of cell cycle data for **(A)** Figure 3D and **(B)** Figure 6A**.**
