# Supplementary material for: Functional RNAi Screening Identifies G2/M and Kinetochore Components as Modulators of TNFα/NF-κB Prosurvival Signaling in Head and Neck Squamous Cell Carcinoma
Source: Cancer Res Commun. 2024 Nov 7;4(11):2903–18. doi: 10.1158/2767-9764.CRC-24-0274 (PMC11541648; doi:10.1158/2767-9764.CRC-24-0274)
Supplement: Supplementary Methods [file crc-24-0274_supplementary_methods_suppsm.docx]

**Supplementary Methods and Materials
siRNA and reagents**siRNA targetting PLK1 (ID:s450), AURKA (ID:A-s195, B-s197), TPR (ID:A-s14353, B-s14354), TTK (ID:A-s120, B-s224757), NUF2 (ID:A-s37982, B-s37983) and NDC80 (ID:s20351) and negative control siRNA (ID:4390847) were purchased from ThermoFisher Scientific (Ambion™ Silencer™ Select siRNA). siRNA was used at 50nM. The small molecule inhibitors Bay1217389 (B389; HY-12859) was purchased from MedChemExpress. All drugs were used at the indicated concentrations and incubation periods listed in the figure legend. Human recombinant TNFα (210-TA) was purchased from R&D Systems and used at 20 ng/μL.

**β-lactamase NF-κB reporter assay**A stable reporter line, UMSCC1^κB^ cells previous developed in our lab (5), were treated as indicated in the figure legends. Relative NFκΒ activity (blue/green fluorescent ratio) was then observed via plate reader.

**Western Blotting**Cells were treated as indicated in the figure legends. Cells were washed with cold 1X PBS, trypsinised and lysed in lysis buffer (10 mM Tris, pH7.5, 0.5% Triton X-100, 150 mM NaCl, 0.5 mM EDTA) containing protease and phosphatase inhibitor cocktail (HALT protease and phosphatase inhibitor cocktail, ThermoFisher Scientific). Protein concentrations were determined using the Pierce BCA Protein Assay Kit (ThermoFisher Scientific). Lysates for each lane were then loaded on SDS-PAGE gels and transferred to nitrocellulose membranes using the Invitrogen iBlot 2 system, according to the manufacturer’s standard protocol. After blocking in Odyssey blocking buffer (Li-COR Biosciences, USA), membranes were incubated with primary antibodies overnight at 4°C. After washing, membranes were incubated with species-specific IRdye-conjugated secondary antibodies for 1 hour at room temperature. Signal was visualized using LI-COR ODYSSEY Infrared Imaging System (Li-COR Biosciences). The following antibodies were used at 1:1000 dilution unless otherwise stated: PLK1 (ab189139, Abcam), AURKA (ab52973), TTK/MPS1 (1:500, ab11108), TPR (ab170940), NUF2 (ab176556), NDC80/HEC1 (1:500; 9G3, ab3613) and β-actin (1:5000, sc-47778, SCBT).

**XTT viability and cell growth assays**

Cells were treated as indicated in the figure legends. For XTT viability assay, XTT reagent was added per the manufacturer’s instructions 48 hours after treatment and plates were read at 450 nm on a plate reader. For growth curves, cells were reseed in 12 well plates 48 hours after treatment. Cell numbers were then counted every 24 hours for 5 days. For each experiment, each condition was performed in triplicate.

**Colony formation assays**
Cells were seeded in 6-well plates and treated as described in the figure legends. For colony formation assays, at the end of treatment, cells were trypsinised and reseeded in a six-well plate at 500 cells per well and left to form colonies for 10-14 days. Colonies were then stained (1% crystal violet, 25% methanol) and were counted manually. Each condition was performed in triplicate.
For clonogenic survival assays, cells were treated with B389 2h prior, or transfected with TTK siRNA 72h prior, to irradiation. Cells were then immediately trypsinised and reseeded in 12-well plates at 100-15000 cells per well (depending on radiation dose) and left to form colonies for 10-14 days. Colonies were then stained (1% crystal violet, 25% methanol) and colonies of ≥50 cells were counted manually. Each condition was performed in triplicate. After correcting for plating efficiency and drug toxicity, survival data were plotted using the linear quadratic model. Radiation dose modifying factors (DMFs) were determined at 10% survival levels by dividing the radiation dose for control by the radiation dose for drug treated. DMFs > 1.0 indicate enhancement of radiosensitivity.
 **Immunofluorescent analysis by confocal microscopy**Cells were seeded onto coverslips. After overnight incubation, cells were treated as required. At the required time, cells were fixed with 4% paraformaldehyde for 10 min and then permeabilized with 0.1% (v/v) Triton for 15 minutes. Cells were blocked in PBS containing 5% BSA for 1 hours. Cells were then incubated in primary antibodies in PBS with 1% BSA overnight at 4°C. Primary antibodies against RELA (8242, CST) or γH2AX (9718, CST) were used at a concentration of 1:400. Cells were washed thoroughly in PBS and then incubated with Alex-fluor conjugated secondary antibodies 594 or Alexa 488 (1:1000; Invitrogen) in PBS with 4% BSA for 2 hours. DAPI was used to visualize nuclei. Coverslips were mounted onto slides with Prolong Gold (Invitrogen). Quantification of nuclear localization and γH2AX foci was quantified and analysed as described (6). Quantification of micronuclei was performed by counting individual nuclei and associated micronuclei per nucleus.

For analysis of mitoses, cells were incubated in Phospho-Histone H3 (Ser10) (D2C8; 3465, CST) and α-Tubulin (11H10; 5059, CST in PBS with 1% BSA overnight at 4°C. DAPI was used to visualize nuclei. Coverslips were mounted onto slides with Prolong Gold (Invitrogen). Individual mitotic cells (as judged by high Phospho-Histone H3 (Ser10), appearance of mitotic spindle and condensed DAPI signal) were then scored based on their phenotypic appearance as: normal, misaligned chromosomes, lagging chromosomes, mono/multi-spindle formation or anaphase bridge formation.

**Flow cytometry assays**
For cell cycle analysis, cells were treated as indicated in the figure legends. At each time point, cells were then harvested by trypsinization, collected by centrifugation, and processed by following the protocol provided by Cycletest Plus DNA Reagent Kit (BD Biosciences). Analysis was performed using a FACS Canto flow cytometer (BD Biosciences). Data from 10,000 cells per sample analyzed using Flow-Jo analysis software (Tree Star). Each condition was performed in triplicate. For γH2AX analysis, cells were treated as indicated in the figure legends. At each time point, cells were tryspsinised, collected by centrifugation and fixed in 4% paraformaldehyde. Cells were then washed in 1% BSA in PBS and permeabilized in 90% methanol. Cells we then incubated with a PE conjugated γH2AX antibody (1:100; 5763, CST) prior to analyzing on a FACS Canto flow cytometer (BD Biosciences). Gates were established using an anti-rabbit IgG negative control. Data from 10,000 cells per sample were analyzed using Flow-Jo analysis software (Tree Star).

For Annexin V assay, cells were treated as indicated in the figure legends. At each time point, cells were then harvested by trypsinization and collected by centrifugation. For UMSCC1 cells, cells were incubated in 100 μL Annexin V staining solution (TACS Annexin V kit; 4830-250-K) for 15 min at room temperature in the dark. Samples were diluted in 1× binding buffer before analysis by flow cytometry on a FACS Canto flow cytometer (BD Biosciences). For UMSCC47 cells, cells were washed in PBS and were then incubated with an Alexa Fluor^®^ 488 conjugated phosphatidylserine (PS) antibody (1:100; 16-256, Sigma-Aldrich). Cells were then stained with propidium iodide (PI) for 30 mins prior to being fixed in 4% paraformaldehyde. Cells were then analyzed on a FACS Canto flow cytometer (BD Biosciences). Data from 10,000 cells per sample analyzed using Flow-Jo analysis software (Tree Star). Early apoptotic cells were defined as Annexin positive/PI negative and late apoptotic cells was defined as Annexin positive/PI positive. Each condition was performed in triplicate.

**Radiation experiments**Cells were irradiated with separate XRAD320 X-ray irradiators (Precision X-ray, Inc., North Branford, CT) housed in the Radiation Biology Branch of the National Cancer Institute. For each experiment, ionizing radiation was delivered at a dose rate of ~2.42 Gy/min with 300kV X-rays at a distance of ~50cm from the radiation source.

**Analyses of publicly available datasets**Analysis of gene and protein expression in HNSCC cases was performed using publicly available data from TCGA (<https://portal.gdc.cancer.gov/repository>; 11), the Clinical Proteomic Tumor Analysis Consortium (NCI/NIH; CPTAC, [https://cptac-data-portal.georgetown.edu](https://cptac-data-portal.georgetown.edu/); 48), DepMap Public 20Q2 release (<https://depmap.org/portal/>) and the Gene Expression Omnibus (GEO; GSE6791 and GSE25099).
